# Jets Downstream of Collisionless Shocks: Recent Discoveries and Challenges

**DOI:** 10.1007/s11214-024-01129-3

**Published:** 2024-12-27

**Authors:** Eva Krämer, Florian Koller, Jonas Suni, Adrian T. LaMoury, Adrian Pöppelwerth, Georg Glebe, Tara Mohammed-Amin, Savvas Raptis, Laura Vuorinen, Stefan Weiss, Niki Xirogiannopoulou, Martin Archer, Xóchitl Blanco-Cano, Herbert Gunell, Heli Hietala, Tomas Karlsson, Ferdinand Plaschke, Luis Preisser, Owen Roberts, Cyril Simon Wedlund, Manuela Temmer, Zoltán Vörös

**Affiliations:** 1 Department of Physics, Umeå University, Linnaeus väg 24, Umeå, 90736 Umeå Sweden; 2 Institute of Physics, University of Graz, Universitätsplatz 5, Graz, 8010 Austria; 3 Department of Physics and Astronomy, Queen Mary University of London, Mile End Road, London, E1 4NS UK; 4 Department of Physics, University of Helsinki, Pietari Kalmin katu 5, 00014 University of Helsinki, Finland; 5 Department of Physics, Imperial College London, South Kensington Campus, London, SW7 2AZ UK; 6 Institute of Geophysics and Extraterrestrial Physics, Technische Universität Braunschweig, Universitätsplatz 2, Braunschweig, 38106 Germany; 7 School of Earth and Atmospheric Sciences, Georgia Institute of Technology, 311 Ferst Drive, Atlanta, 30332 GA USA; 8 KTH Royal Institute of Technology, Department of Space and Plasma Physics, School of Electrical Engineering and Computer Science, Teknikringen 31, Stockholm, 100 44 Sweden; 9 Johns Hopkins University, Applied Physics Laboratory, 11000 Johns Hopkins Rd, Laurel, 20723 MD USA; 10 Department of Physics and Astronomy, University of Turku, Vesilinnantie 5, Turku, 20014 Finland; 11 Faculty of Mathematics and Physics, Charles University, V Holešovičkách 2, Prague, 180 00 Czech Republic; 12 Instituto de Geofísica, Universidad Nacional Autónoma de México, Circuito de la Investigación Científica s/n, México City, 04150 CDMX Mexico; 13 Space Research Institute, Austrian Academy of Sciences, Schmiedlstraße 6, Graz, 8042 Austria; 14 Department of Physics, Aberystwyth University, Physical Sciences Building, Aberystwyth, SY23 3BZ UK; 15 Institute of Earth Physics and Space Science, HUN-REN, Csatkai E. u. 6-8., Sopron, 9400 Hungary

**Keywords:** Magnetosheath jets, Magnetosheath, Foreshock, Solar wind, Bow shock, Magnetopause

## Abstract

Plasma flows with enhanced dynamic pressure, known as magnetosheath jets, are often found downstream of collisionless shocks. As they propagate through the magnetosheath, they interact with the surrounding plasma, shaping its properties, and potentially becoming geoeffective upon reaching the magnetopause. In recent years (since 2016), new research has produced vital results that have significantly enhanced our understanding on many aspects of jets. In this review, we summarise and discuss these findings. Spacecraft and ground-based observations, as well as global and local simulations, have contributed greatly to our understanding of the causes and effects of magnetosheath jets. First, we discuss recent findings on jet occurrence and formation, including in other planetary environments. New insights into jet properties and evolution are then examined using observations and simulations. Finally, we review the impact of jets upon interaction with the magnetopause and subsequent consequences for the magnetosphere-ionosphere system. We conclude with an outlook and assessment on future challenges. This includes an overview on future space missions that may prove crucial in tackling the outstanding open questions on jets in the terrestrial magnetosheath as well as other planetary and shock environments.

## Introduction

This review summarises recent studies on magnetosheath jets: transient mesoscale plasma entities characterised by high dynamic pressures, observed downstream of collisionless shocks. In particular, this review builds on and extends that of Plaschke et al. (2018), which comprehensively catalogued our knowledge of jets from their initial discovery up to the time of its writing. As such, this article focuses on recent discoveries not covered by those authors, which we define as those published since 2016. We discuss how our interpretation of jets has developed in this time, and make suggestions for the future. In the remainder of this section, we introduce the contexts in which jets are observed and summarise their more well-established attributes, briefly outlining the key studies that shaped our understanding. We then outline the article structure.

**Fig. 1:**
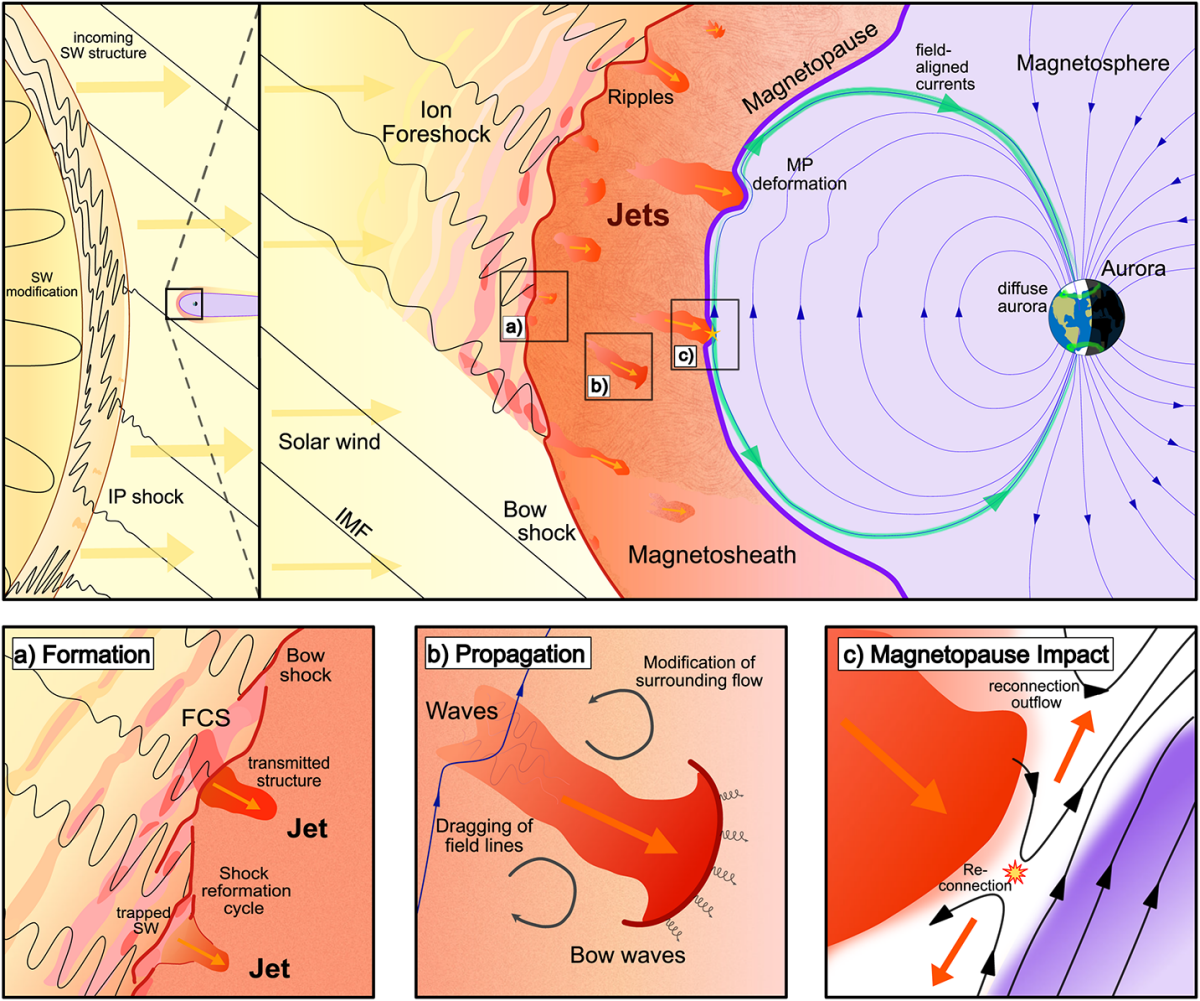
A sketch of the dayside plasma environment of the Earth. Several jets are depicted in the magnetosheath between the solar wind (coming from the left) and Earth’s magnetosphere (right). The system is influenced by the solar wind and its structures such as interplanetary (IP) shocks, one of which is depicted in the top left panel. Most jets are formed at the quasi-parallel shock front as a result of the shock’s non-stationary processes such as rippling, or the interaction with foreshock compressional structures (FCSs). This is depicted in Panel a). Jets then propagate into the turbulent magnetosheath while modifying the surrounding plasma and field structure, sometimes launching bow waves (Panel b)). Some jets survive the magnetosheath crossing and impact the magnetopause, where they may induce dayside reconnection (Panel c)). Secondary effects of impacting jets like magnetopause deformation, field aligned currents, and diffuse aurora are also depicted in the global sketch

The solar wind is a continuous plasma flow originating at the Sun and extending across space. When it encounters Earth’s magnetic field, which acts as an obstacle, the interaction shapes a complex environment with dynamics highly dependent on the solar wind properties. The region where the plasma motion is controlled by the Earth’s intrinsic magnetic field is known as the magnetosphere (depicted as a purple region in Fig. 1). The magnetosphere’s outermost boundary is the magnetopause where the dynamic pressure of the solar wind and the magnetosphere’s magnetic pressure are balanced (purple line in Fig. 1). At Earth’s orbital distance, the solar wind is supermagnetosonic and forms a bow shock in front of the Earth’s magnetosphere. The bow shock is a collisionless shock front where the solar wind plasma is decelerated, heated, and compressed (dark red line in Fig. 1). The region between the bow shock and the magnetopause is called the magnetosheath (light red region in Fig. 1) where the shocked solar wind plasma is deflected around the magnetosphere.

The bow shock structure can be characterised by $\theta _{Bn}$, the angle of the interplanetary magnetic field (IMF) with respect to the shock normal vector. The bow shock is referred to as ‘quasi-perpendicular’ where $\theta _{Bn} > 45^{\circ}$ and ‘quasi-parallel’ where $\theta _{Bn} < 45^{\circ}$. The term ‘oblique’ is sometimes used for representing intermediate values close to 45^∘^. In the magnetosheath, close to the subsolar point, the IMF cone angle (the angle between the IMF and the Sun–Earth line) is sometimes used as a proxy for $\theta _{Bn}$. The quasi-perpendicular bow shock has a well-defined structure with a foot, ramp and an overshoot (Bale et al. 2005, and references therein). In contrast, the quasi-parallel bow shock shows a more complex behaviour as a fraction of the incoming particles are able to return back upstream, interact with the incoming solar wind, and generate a turbulent region upstream of the bow shock called the foreshock (Eastwood et al. 2005). In this region, various transient structures (see a complete review by Zhang et al. 2022), such as short large amplitude magnetic structures (SLAMS) (Schwartz et al. 1985), hot flow anomalies (HFAs) (Schwartz et al. 1985), shocklets (Hoppe et al. 1981), cavitons (Lin 2003; Omidi and Sibeck 2007; Blanco-Cano et al. 2009), foreshock bubbles (Omidi et al. 2010; Turner et al. 2013), and magnetic holes (Turner et al. 1977), can be generated locally. Downstream of the bow shock, in the magnetosheath, different plasma properties are seen depending on whether a region is magnetically connected to the quasi-parallel or quasi-perpendicular bow shock (Raptis et al. 2020a; Karlsson et al. 2021). The region downstream of the quasi-parallel bow shock is generally more turbulent compared to the magnetosheath downstream of the quasi-perpendicular bow shock (Formisano and Hedgecock 1973; Luhmann et al. 1986; Raptis et al. 2020a).

**Fig. 2:**
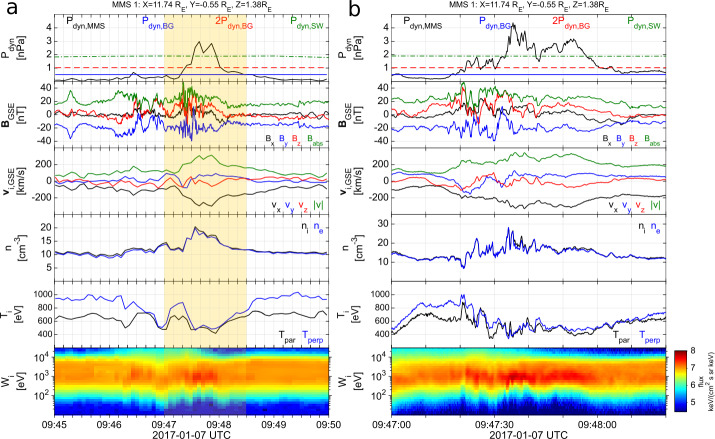
Example of a magnetosheath jet using (a) the fast and (b) the burst data of MMS1. The yellow shaded area of (a) represents the time interval shown in (b). [Top-Bottom]: Ion dynamic pressure with different thresholds based on background magnetosheath values and upstream solar wind measurements, magnetic field components and magnitude in GSE coordinates, ion velocity in GSE coordinates, ion and electron density, ion temperature parallel and perpendicular to the field, and ion differential energy spectrum. MMS position in GSE coordinates is shown at the top of the figure

Transient dynamic pressure enhancements, knows as magnetosheath jets, are often observed in the magnetosheath (illustrated in Fig. 1 as orange structures). An example of a magnetosheath jet observed by the Magnetospheric Multiscale mission (MMS) is shown in Fig. 2. Jets are found to occur more often downstream of the quasi-parallel bow shock than the quasi-perpendicular shock (e.g. Archer and Horbury 2013; Plaschke et al. 2013; Vuorinen et al. 2019; Raptis et al. 2020a). Most jets are thought to form at the bow shock and propagate through the magnetosheath towards the magnetopause. Their increased occurrence rate downstream of the quasi-parallel bow shock links jets to foreshock processes, however the dominant generation mechanism is still under debate. In the magnetosheath, jets interact with the surrounding plasma, often dissipating during their travel. Finally, a fraction of jets impact the magnetopause (Hietala et al. 2009) and cause disturbances inside the magnetosphere (Gunell et al. 2012). This implies that jets can be drivers of the coupling between the solar wind and the magnetosphere-ionosphere system, transporting energy from the foreshock and bow shock into the magnetosphere. The stages of formation, propagation, and impact of magnetosheath jets are illustrated in Fig. 1. In Plaschke et al. (2018) the authors provide a comprehensive review of the literature concerning jets up to 2017. In addition, Echim et al. (2023) discussed findings using theoretical descriptions of plasma irregularities propagating through the magnetosheath. With many recent advancements in the field, it has become necessary to extend these collections. Therefore, in this review, we will present and discuss new findings concerning magnetosheath jets that have been published since 2016.

Recent advancements in spacecraft instrumentation and computational simulations have brought new insights into the formation, propagation, and magnetospheric effects of magnetosheath jets. For example, the Magnetospheric Multiscale (MMS) mission (Burch et al. 2016), which was launched in 2015, provided unprecedented time resolution on its scientific payload. This has allowed for new investigations concerning the internal properties of jets and their interactions with the ambient plasma. Figure 2b shows a magnetosheath jet observed in MMS burst mode where the micro-structure is well-resolved compared to Fig. 2a. Furthermore, the spacecraft constellation, with its smaller spacecraft separation than that of the Cluster mission, has allowed for new insights into jet formation mechanisms. For example, Raptis et al. (2022b) recently reported the formation of a jet due to shock reformation. The Time History of Events and Macroscale Interactions during Substorms (THEMIS) mission (Angelopoulos 2008), has now collected data for the entirety of solar cycle 24 which makes it possible for jets, their characteristics, dynamics, distribution, and dependence on solar wind upstream conditions, to be studied from a statistical point of view. In addition, steady progress has been made in numerical simulations which has improved our understanding of formation mechanisms, properties, and propagation of jets from global (e.g., Palmroth et al. 2018; Omelchenko et al. 2021; Suni et al. 2021, 2023) and local perspectives (e.g., Hao et al. 2016; Preisser et al. 2020; Tinoco-Arenas et al. 2022). Recently, Omelchenko et al. (2021) presented the first study on magnetosheath jets using a global 3D Hybrid simulation and Palmroth et al. (2018) demonstrated how Vlasiator, an unscaled hybrid-Vlasov simulation, can be used for magnetosheath jet research. Vlasiator is a 3D simulation but has so far been restricted to 2D for jet research.

It is important to note that the naming and definition of magnetosheath jets is not consistent in the literature. When first described, jets were referred to as ‘transient flux enhancements’ (Němeček et al. 1998). Other terms such as ‘dynamic pressure pulses’ (Archer et al. 2012), ‘high speed jets’ (Plaschke et al. 2013), or ‘plasmoids’ (Gunell et al. 2014; Karlsson et al. 2015) can also be found in the literature. Similarly, different methods have been used for the identification of magnetosheath jets. As jets are defined as enhancements of a physical quantity, imposing a threshold is required. The common approach is to compare the local plasma dynamic pressure either to the background magnetosheath plasma or with the solar wind dynamic pressure. A detailed comparison between different criteria used for magnetosheath detection and naming of jets can be found in Plaschke et al. (2018). In this article we will refer to these structures as ‘magnetosheath jets’ or, simply, ‘jets’.

Jet formation is connected to fundamental processes occurring at collisionless shocks caused by the dynamics of the environment, as well as discontinuities interacting with the shock (see Fig. 1). Studying the formation mechanisms of jets is therefore essential for understanding the variability of collisionless shocks in general. Plaschke et al. (2018) discussed jet formation from foreshock structures that cause shock rippling, solar wind discontinuities, cavitons, and spontaneous hot flow anomalies. The authors also reported that $\theta _{Bn}$ is highly linked to predicting jet formation. New findings concerning formation mechanisms are discussed in Sect. 2. Using the Vlasiator simulation, Suni et al. (2021) revealed the importance of the foreshock for the formation of jets. Moreover, this section discusses new insights in jet occurrence rates which have been shown not to be solely dependent on the IMF cone angle (LaMoury et al. 2021; Vuorinen et al. 2023a). Furthermore, the occurrence rates of jets during the passage of large-scale solar wind structures was investigated by Koller et al. (2022) and Koller et al. (2023). In addition, for the first time, jets have been observed at other planetary magnetosheaths (Gunell et al. 2023; Zhou et al. 2024) and at interplanetary shocks (Hietala et al. 2024).

As jets propagate through the magnetosheath, the plasma in front of the jet is compressed and the magnetosheath flow around the jets disturbed, contributing to local turbulence. In addition, jets interact with the ambient plasma giving rise to instabilities and wave generation as depicted in Fig. 1b. Plaschke et al. (2018) discussed properties of jets such as their morphology and the variability of the magnetic field and plasma moments. In recent years, jets have been associated with non-Maxwellian distribution functions indicating the mixing of two particle populations (Raptis et al. 2022a). There are new findings about waves inside and in the vicinity of jets (Karlsson et al. 2018; Blanco-Cano et al. 2020; Krämer et al. 2023). Observed jet properties and new understanding concerning their evolution through the magnetosheath is discussed in Sect. 3.

Jets can impact the magnetopause and subsequently cause disturbances in the magnetosphere and ionosphere. Under low $\theta _{Bn}$ IMF conditions, jets are known to frequently impact the magnetopause (Plaschke et al. 2020b). Furthermore, there is an ongoing discussion about the role of jets in space weather. Plaschke et al. (2018) discussed a possible connection between throat aurora and magnetosheath jets. In addition, the authors raised the possibility that jets might trigger or suppress reconnection (see Fig. 1), based on simulation results (Karimabadi et al. 2014). In Sect. 4 we discuss observational evidence strengthening the connection between jets and auroral signatures (Wang et al. 2018; Nishimura et al. 2020). Moreover, we discuss recent studies and observational evidence that jets can trigger magnetic reconnection (Hietala et al. 2018) and subsequently contribute to the onset of a magnetospheric substorm (Nykyri et al. 2019). Additionally, jets have been shown to trigger magnetopause surface waves (Archer et al. 2019). Recent developments about the role of jets and ground-based of ultra-low frequency waves are also presented.

Finally, in Sect. 5 we present outstanding open questions, as well as an outlook, and suggestions for the future. We include a discussion of how future spacecraft missions will support research on magnetosheath jets, both at Earth and at other collisionless shock environments.

## Jet Occurrence and Formation

The ever-increasing number of observations, recent advances in numerical kinetic simulations, and more comprehensive analyses have significantly contributed to our understanding of jet formation. This section provides a review of the latest developments in the study of jet occurrence and the insights gained into their formation through statistical studies, case studies, and simulations. Jet formation mechanisms continue to be a subject of ongoing research (Preisser et al. 2020; Raptis et al. 2022b; Zhou et al. 2023; Suni et al. 2021, 2023). Many recent studies have focused on the formation of jets in close association with the quasi-parallel bow shock, reflecting the fact that the majority of jets appear to originate there (Vuorinen et al. 2019; Raptis et al. 2020a; LaMoury et al. 2021; Tinoco-Arenas et al. 2022; Koller et al. 2023). In addition, the first evidence of jets in environments other than the terrestrial magnetosheath has recently been reported (Gunell et al. 2023; Zhou et al. 2024; Hietala et al. 2024).

### Occurrence and Dependence on Solar Wind

The following subsections examine latest results on the influence of solar wind parameters on jet occurrence and the spatial location of jets within the magnetosheath. Both topics are intertwined and give context to jet propagation, which will be examined in more detail in Sect. 3.2.3. We also review the first-ever reports on jet detection outside of the terrestrial magnetosheath.

Several works estimated the occurrence rates of jets with a focus on impacts at the magnetopause. We refer to Sect. 4.1 for an overview on jet occurrence rates at the magnetopause.

#### Jet Occurrence Statistics Based on Solar Wind Parameters

The origin of magnetosheath structures is a major topic of ongoing investigation in Earth’s magnetosphere. Numerous previous studies (e.g. Karlsson et al. 2015; Hietala and Plaschke 2013; Suni et al. 2021; Raptis et al. 2022b) connected the formation of jets in the magnetosheath with the existence of upstream foreshock plasma and transients within it. The system comprising the foreshock, bow shock, and magnetosheath is therefore highly dependent on the solar wind parameters. This makes the upstream solar wind the major factor of influence to jet generation in the magnetosheath (Vuorinen et al. 2019; Goncharov et al. 2020; Raptis et al. 2020a; LaMoury et al. 2021; Koller et al. 2023).

In the earliest studies on jets, called ‘transient flux enhancements’ by Němeček et al. (1998), they found that the occurrence in the flank magnetosheath depended on there being steady solar wind with $M_{A} \sim 7$. The statistical studies by Archer and Horbury (2013), Plaschke et al. (2013) and Gutynska et al. (2015) concluded that jet occurrence increases when the IMF is steadier than usual, resulting in stable foreshock processes at the quasi-parallel shock. Archer and Horbury (2013) found that the angle $\theta _{Bn}$ is the most important parameter for jet formation. Plaschke et al. (2013) came to the same result, reporting that the IMF cone angle is the only significant parameter for jet occurrence in the subsolar region. The latter study also noted a minor increase during increased solar wind velocity, Alfvénic Mach number, and lower density, which themselves are related to small IMF cone angles. While steady solar wind is preferable for the majority of jets, a minority exist which appear associated with solar wind discontinuities and cannot be explained by chance (Archer and Horbury 2013), in agreement with several case studies on discontinuity-related jets (e.g. Archer et al. 2012; Savin et al. 2012). All these studies are summarised in the review paper by Plaschke et al. (2018).

In previous case-based and statistical works, various jet identification criteria have been used (see Table 1 in Plaschke et al. 2018). Recent studies connecting jets to solar wind parameters have often used the criteria by Plaschke et al. (2013) or a modified version (Vuorinen et al. 2019; Goncharov et al. 2020; LaMoury et al. 2021; Koller et al. 2022). The criterion introduced by Plaschke et al. (2013) defines jets as $P_{\mathrm{dyn,MSH},x}>0.5P_{\mathrm{dyn,SW}}$, thus, only earthward dynamic pressure increases are considered. Additionally, only flows with a 50% increase in earthward velocity are considered. The aforementioned studies have considered the upstream solar wind state to evaluate which conditions may drive the generation of these jets. These studies focused on jets that are observed in the subsolar region due to their higher potential to impact the magnetopause. However, Goncharov et al. (2020) also included a small number of observations from the magnetosheath flanks. Koller et al. (2022, 2023) also used a modified version of the Archer and Horbury (2013) criterion based on the local 20-minute-averaged magnetosheath dynamic pressure, where $P_{\mathrm{dyn,MSH},x}>3\langle P_{\mathrm{dyn,MSH},x}\rangle _{ \mathrm{20min}}$. Lastly, Goncharov et al. (2020) imposed a modified Plaschke et al. (2013) condition where $P_{\mathrm{dyn,MSH},x}>P_{\mathrm{dyn,SW}}$ and the jet interval is defined by $P_{\mathrm{dyn,MSH},x}>0.4P_{\mathrm{dyn,SW}}$. We compare these studies because they use similar criteria, the same plasma parameter, and are narrowed down to the same subsolar magnetosheath region. Studies utilising upstream solar wind monitors primarily make use of the OMNI dataset (King and Papitashvili 2005), which propagates solar wind measurements close to the L1 point to the nose of the bow shock.

Vuorinen et al. (2019) used THEMIS and OMNI data between 2008 and 2011 in the subsolar magnetosheath. They studied the distribution of jets during different IMF orientations finding that the majority of them are closer to the bow shock than to the magnetopause. Jets appeared 9 times more frequently downstream of the quasi-parallel than the quasi-perpendicular shock. This is in agreement with the general trend found in previous observational studies by Archer and Horbury (2013) and Plaschke et al. (2013) as well as a recent statistical study based on 2D hybrid simulations by Tinoco-Arenas et al. (2022). There was a monotonic increase of jet observations from the quasi-perpendicular side towards the quasi-parallel side. Goncharov et al. (2020), using MMS and OMNI data between 2015 and 2017, characterised magnetosheath jets (and plasmoids as defined in Karlsson et al. 2015, 2016) depending on their position in either the quasi-parallel or the quasi-perpendicular downstream region. They also observed that jets appeared more frequently in the quasi-parallel region. In general, they found the jet occurrence to increase when the solar wind is faster, has increased magnetic field strength, high plasma $\beta $, and increased Alfvénic Mach numbers compared to the overall solar wind distribution. The jets were mostly observed under steady IMF, with 15% being connected to a solar wind discontinuity. This, however, does not fully account for all quasi-perpendicular jets detected in the study. Raptis et al. (2020a) used MMS measurements and found in agreement with previous works that jets occur more frequently in quasi-parallel magnetosheath plasma, while Raptis et al. (2020a,b) found that quasi-parallel jets are potentially associated with higher solar wind velocity compared to the quasi-perpendicular ones. LaMoury et al. (2021) studied magnetosheath jets using THEMIS and OMNI data in the years 2008–2018 which include the data set of Plaschke et al. (2013) and Vuorinen et al. (2019). One difference in the results of this study and Goncharov et al. (2020) is that the former found an increased occurrence rate for low instead of large IMF magnitudes. Notably, the absolute value for favourable jet occurrence in IMF magnitude is still comparable in both studies. Additionally, LaMoury et al. (2021) reported a preference of jet formation at low IMF cone angles and a minor preference in low solar wind densities. An important result of their study is that solar wind parameters control the formation of jets differently than their propagation, and therefore restricted their study of factors influencing occurrence to those observed near the bow shock. This suggests that future studies should take the jet distance to the bow shock into account when discussing jet formation based on solar wind parameter.

Vuorinen et al. (2023a) statistically investigated the influence of solar wind parameters during low and high IMF cone angle regimes in more detail. They found that jet formation commences for SW plasma $\beta $ above 0.5 and solar wind Alfvén Mach number above 5 for both regimes. Jet formation close to the bow shock during low cone angles (behind the quasi-parallel shock) was not significantly influenced by any additional solar wind parameter. However, in high cone angle conditions (behind the quasi-perpendicular shock), the jet occurrence was sensitive to several additional parameters: low IMF magnitude, high solar wind speed, low density, high plasma $\beta $, and high Alfvén Mach number increased the jet occurrence behind the quasi-perpendicular shock. Investigating two example events, they noted that these jets appeared to be a part of the quasi-perpendicular shock dynamics at high plasma $\beta $ and Alfvén Mach numbers, but were only observed close to the shock transition region.

**Fig. 3:**
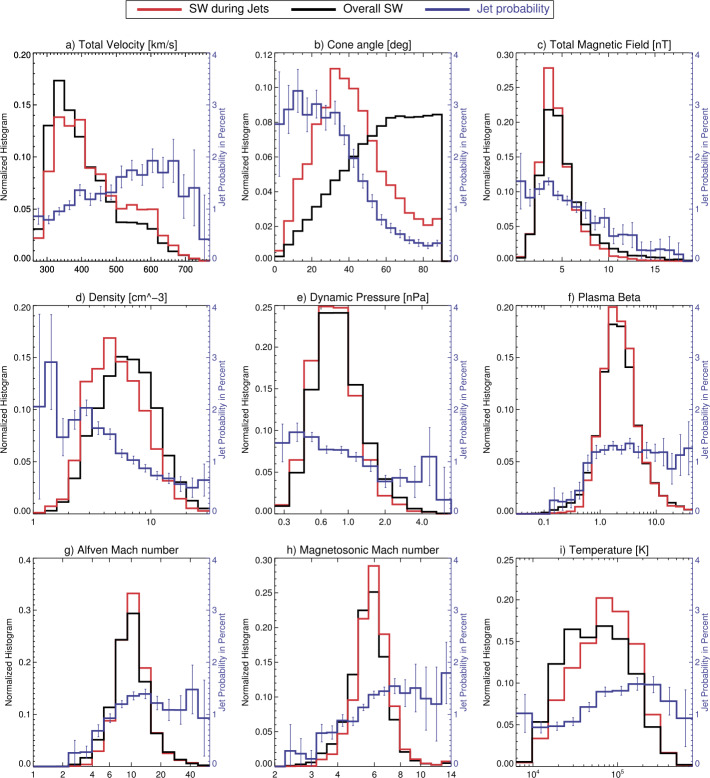
Solar wind parameter distributions during jets (red), all available sheath data (black) and jet probability distribution (blue). Based on THEMIS observations from 2008–2022. Image reproduced with permission from Koller et al. (2023), copyright by the author(s)

Koller et al. (2022) attempted to associate the generation of jets to large-scale solar wind structures, like coronal mass ejections (CMEs), stream interaction regions (SIRs) and high speed streams (HSSs). CMEs are quickly expanding interplanetary transients that posses strong magnetic field strength in their inner, “flux-rope” like structure. HSSs emanate from coronal holes and cause SIRs by interacting with the slow solar wind in front of it (Temmer 2021). Thus, SIRs show compressed plasma in the front with high density and high magnetic field strength, and afterwards high velocities and low densities in the HSSs in the back of SIRs. Koller et al. (2022) used data from THEMIS and OMNI between 2008 and 2020, and combined different lists and catalogues for CMEs, SIRs and HSSs. From the analysis they noticed an increase in jet occurrence (∼20$\%$–50$\%$) for SIRs and HSSs, but a drop or no change under the presence of CMEs. It was discussed that CMEs disturb the foreshock due to significant changes in the IMF, leading to unfavourable conditions for jet formation in the subsolar region. On the other hand, SIRs and HSSs are associated to high fast solar wind conditions that are favourable for jets’ generation, as also stated by Goncharov et al. (2020) and LaMoury et al. (2021). In a follow-up study, Koller et al. (2023) investigated all solar wind parameters and their relation to jet occurrence. Figure 3 shows the solar wind parameter distributions during jets compared to the overall distributions used in Koller et al. (2023). The blue line shows the jet probability distribution defined as jet interval observation time divided by the total observation time for each bin. They verified that CMEs are not favourable for the creation of jets since they are associated with high IMF cone angles and low Alfvén Mach number. HSSs on the other hand have proved to be in favour of jet generation since they are connected to high velocity, low density, high Mach numbers, and, most importantly, statistically a higher chance of low cone angles.

Table 1 summarises the findings of the previously mentioned studies relating solar wind conditions and jet occurrence. These trends agree in general with the findings from previous studies see (see Plaschke et al. 2016). Notably, several parameters in the solar wind are interdependent as shown in a solar wind parameter correlation matrix in Koller et al. (2023). One of the main recent findings on the generation of jets is that the solar wind provides steady conditions for the formation of the foreshock region and thus the quasi-parallel downstream magnetosheath. Another important condition has been shown to be a low IMF cone angle and a fast solar wind, which are also associated with HSSs. It is thus expected that jet occurrence is increased in solar wind plasma with high speed, high Alfvén Mach number, and high plasma $\beta $. The occurrence is weakly dependent on the solar wind density and dynamic pressure, with lower values appearing to be more favourable for jet formation. There is no absolute consensus whether an increase in IMF magnitude (relative to the base solar wind distribution for each study) is favourable for jet formation of jets or not, although the absolute values for jet detection agree in the studies.

**Table 1:** Summary of favourable solar wind conditions for jet occurrence in the subsolar magnetosheath based on five studies. The table lists the following parameters: cone angle, IMF field strength, solar wind velocity, solar wind density, Alfvén Mach number, plasma beta, and ion temperature. *High*, *medium*, and *low* indicate the trend of favourable conditions or the average occurrence peak in relation to the overall solar wind parameter distribution

Study	Cone Angle	IMF *B*	*V*	*n*	$M_{\mathrm{A}}$	*β*	*T*
Vuorinen et al. (2019)	low, ≤30^∘^	-	-	-	-	-	-
Goncharov et al. (2020) ($Q_{\parallel}$)	low, peak ∼20^∘^	medium, ∼5.89 nT	high, ∼492 km/s	–	high, ∼9.8	high, ∼0.87	–
LaMoury et al. (2021)	low, <30^∘^	low, <6nT	high, >500 km/s	low, <3.5 cm^−3^	high, >7	high, >1	–
Koller et al. (2022)	low	–	high	low	high	–	high
Koller et al. (2023)	low, <40^∘^	low, <6 nT	high, >500 km/s	low, <4 cm^−3^	high, >7	high, >0.5	high, >6 × 10^4^ K

The occurrence of solar wind structures like HSSs and CMEs changes significantly within a solar cycle. Motivated by the results of these structures modifying the occurrence of jets, Vuorinen et al. (2023b) analysed the change of jet occurrence over solar cycle 24 (2008-2019) using THEMIS measurements. They recognised that several factors can introduce biases in the long-term statistical data. The spacecraft orbit apogee can introduce a significant bias, because more jets are measured when the spacecraft is closer to the bow shock. In order to produce an unbiased result, Vuorinen et al. (2023b) constructed a model that predicted the occurrence of jets in the dayside magnetosheath close to the bow shock based on solar wind parameters. A model using the IMF cone angle and IMF magnitude only, gave the best estimates of jet occurrences for each time instance. This model was then used on the whole distribution of the solar wind parameters over the solar cycle 24 as well as solar cycle 23. The results showed that the solar cycle only slightly modifies the jet occurrence: the number of jets decrease by roughly 10–20% during solar maximum conditions. This was attributed to the higher number of CMEs occurring during the maximum. Notably, this decrease is also comparable to the error margin of the model.

In addition to the above mentioned observational studies, advances have been made possible by numerical simulations to infer the influence of solar wind parameters on the occurrence of jets. In a statistical study, Tinoco-Arenas et al. (2022) examined jet properties as a function of $\theta _{Bn}$ and upstream velocity $V_{in}$ based on a large set of 2D local hybrid simulations of collisionless shocks. Their runs explored a variety of IMF angles (15^∘^ $\leq \theta _{Bn} \leq $ 65^∘^) and low to intermediate Alfvén Mach numbers (4.28 $\leq M_{A} \leq $ 7.42) at the shock. Four different observation-based criteria were used for the identification of jets: high-speed jets (Plaschke et al. 2013), transient flux enhancements (Němeček et al. 1998), density plasmoids (Karlsson et al. 2015), and high-speed plasmoids (Gunell et al. 2014) (see Plaschke et al. 2018, for an overview on these criteria). The number of structures in the simulations depended on the used criteria with transient flux enhancements being the most numerous, followed by high-speed jets, density plasmoids, and lastly high-speed plasmoids. They found that density plasmoids were produced only by shocks with $M_{A}\geq 5.7$, while the high-speed plasmoids only formed downstream of shocks with $M_{A}\geq 6.97$. The simulations showed that higher-$M_{A}$ shocks tended to produce jets with higher velocities and larger surface area, mass, linear momentum, and kinetic energy. These properties appeared to be anti-correlated with $\theta _{Bn}$. The increase of $\theta _{Bn}$ up to 45^∘^ resulted in an increment of jet production. On the other hand, jet production ceased for $\theta _{Bn}=$ 65^∘^ independently of the shock’s $M_{A}$. Using 2D global hybrid simulations Guo et al. (2022) investigated the formation and evolution of jets with different solar wind conditions. They found that the alignment of magnetic field and velocity in the upstream side of the bow shock favours the formation of large-scale jets (size of about $2.5 R_{E}$) at the magnetosheath where $\theta _{Bn}\sim \,0^{\circ}$.

To conclude this subsection: while the dependence of jet occurrence with the IMF angle has been further clarified, we have also gained insights on the dependence on other solar parameters and consequently, solar wind structures. A major takeaway is that solar wind parameters influences formation and propagation of jets differently (LaMoury et al. 2021). Thus, when analysing jet formation in observations, a restriction to near-shock regions may be necessary (also discussed for spacecraft orbit biases by Vuorinen et al. 2023b).

#### Spatial Occurrence Within the Magnetosheath

As discussed in the previous section, it has been well established that magnetosheath jets predominantly occur behind the quasi-parallel bow shock with a higher occurrence rate close to the bow shock than to the magnetopause (Plaschke et al. 2018, and references therein). Recently, a greater availability of statistics and new simulations have further improved our knowledge of the spatial occurrence of jets within the magnetosheath.

**Fig. 4:**
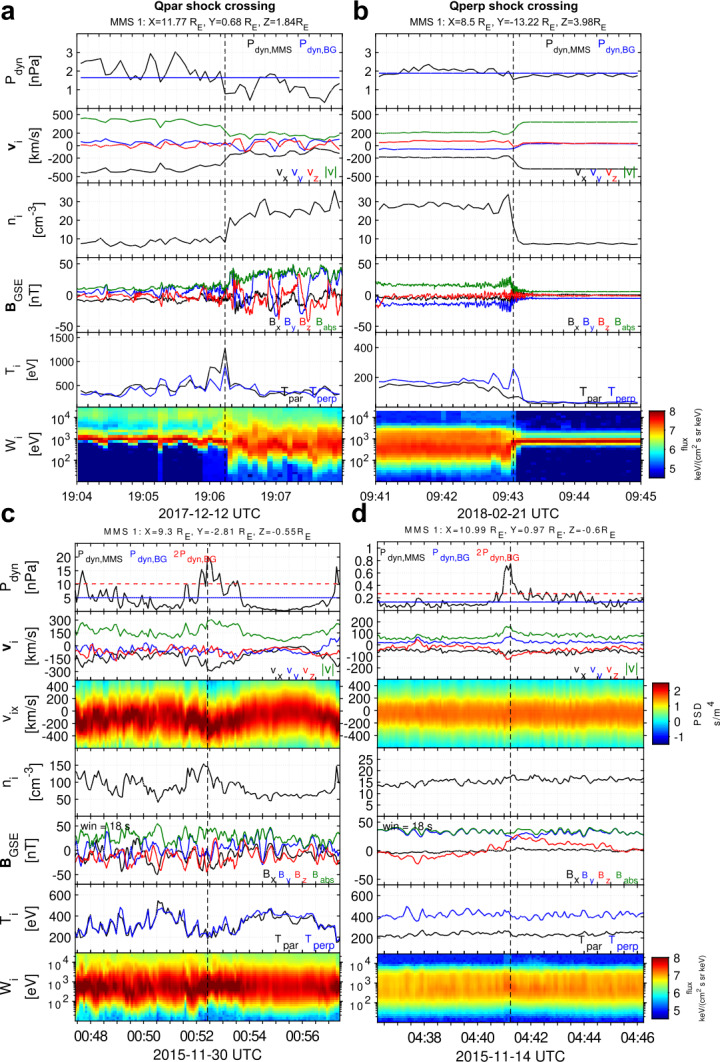
MMS1 measurements showing (**a**) an example of an inbound quasi-parallel bow shock crossing and (**b**) an example of an outbound quasi-perpendicular bow shock crossing. (Top — bottom): ion dynamic pressure, ion velocity, ion number density, magnetic field vector, ion temperature, and ion differential energy spectra. (**c**) shows an example of a jet observed in the quasi-parallel magnetosheath and, (**d**), of a jet in the quasi-perpendicular magnetosheath. (Top — bottom): ion dynamic pressure and background magnetosheath level, ion velocity in GSE coordinates, reduced 1D VDFs in the $x$ GSE direction, ion number density, magnetic field measurements, ion temperature, and ion differential energy spectra. Images adapted from Raptis (2022) in courtesy of S. Raptis

**Fig. 5:**
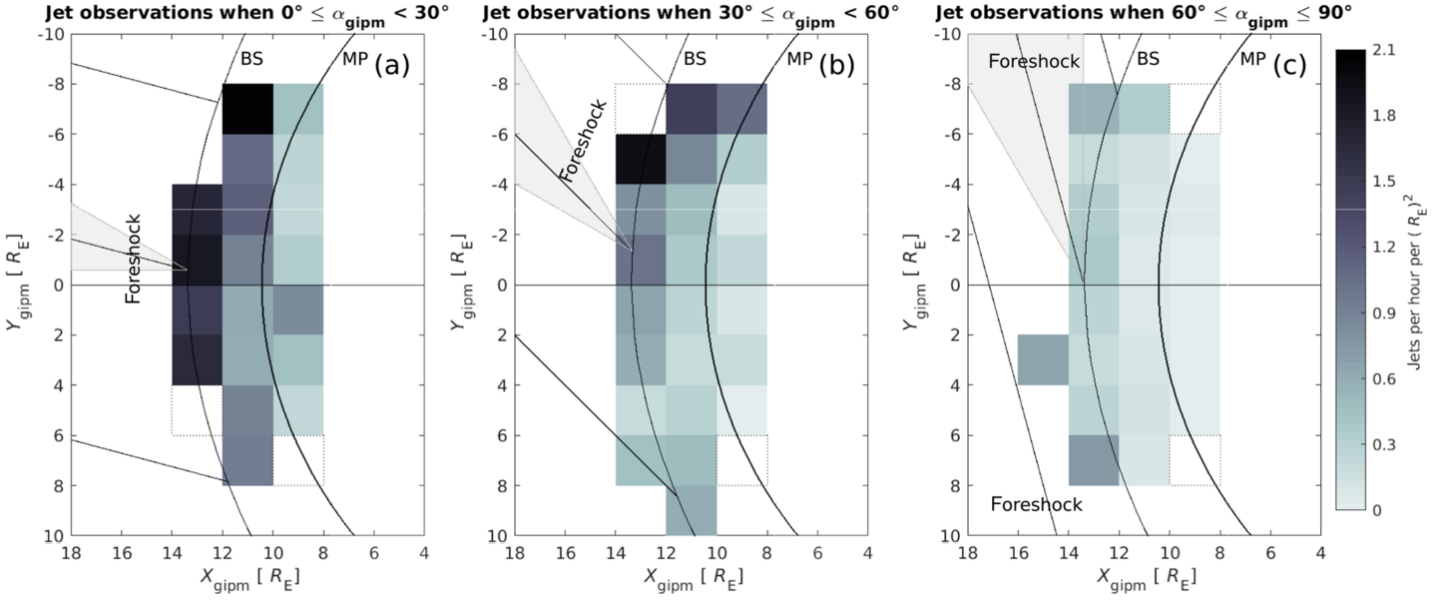
Jet occurrence dependence on distance to bow shock and cone angle based on THEMIS observations from 2008–2011. The maps show the number of jets per hour with respect to the bow shock and magnetopause position under three different cone angle regimes. Most jets appear close to the bow shock in quasi-radial conditions (panel a)). The bow shock and magnetopause models show the position during under the average solar wind dynamic pressure. Images reproduced with permission from Vuorinen et al. (2019), copyright by the author(s)

Recent studies brought improved estimates on the jet occurrence in terms of regions of the magnetosheath – quasi-parallel, quasi-perpendicular or in between. To give context based on observational data by MMS, Fig. 4 shows a spacecraft crossing into the quasi-parallel sheath (a), a crossing from the quasi-perpendicular sheath into the solar wind (b), example jets in the quasi-parallel sheath (c), and in the quasi-perpendicular sheath (d). As mentioned in Sect. 2.1, Vuorinen et al. (2019) found that jets occur approximately 9 times more often in the quasi-parallel magnetosheath. Figure 5 shows the spatial distribution of jet occurrence based on the relative position in the magnetosheath and the cone angle. Despite the clear preference, the statistical analysis by Goncharov et al. (2020) using two years of MMS measurements found that jets behind the quasi-perpendicular bow shock are not uncommon, with most of those jets being connected to oblique IMF angles.

Based on MMS measurements, Raptis et al. (2020a) investigated jets and their properties in different regions of the magnetosheath. They reported a similar relation as Vuorinen et al. (2019) with about 5–10 times more jets in the quasi-parallel compared to the quasi-perpendicular sheath. Raptis et al. (2020a) defined so-called “boundary jets”, which show a different background magnetosheath before and after the jet. These jets appear between the quasi-parallel and quasi-perpendicular magnetosheath regions while showing the properties similar to quasi-parallel jets.

In addition, they found a subset of jets, designated “encapsulated jets”, with quasi-parallel properties that were detected within the quasi-perpendicular sheath and were found on average further away from the bow shock. Raptis et al. (2020a) discussed possible origins of these jets: a subset was attributed to plasma reflection at the magnetopause showing sunward motion. For jets with dominant earthward motion, it was proposed that the jets form close to a region containing an IMF rotation and then migrate from the quasi-parallel to the quasi-perpendicular region due to their increased speed compared to their surroundings. For most other encapsulated jets, these transients were assumed to form at the flanks of the bow shock during times of non-radial IMF. The jets then migrate inward and into the quasi-perpendicular region. In a complementary work, the classification of jets into different regions (quasi-parallel, quasi-perpendicular, boundary, encapsulated) was conducted using Neural Networks and high-resolution OMNI data by Raptis et al. (2020b), confirming the previous results. Koller et al. (2024) pointed out that magnetosheath classification might become inaccurate under fast solar wind conditions if not corrected for the solar wind input, which can heavily affect the results on spatial occurrence of jets.

Recent studies also brought new insights to the radial distance of jets from the bow shock to the magnetopause. This distance can be used as a proxy how far jets propagate into the magnetosheath. Using THEMIS and MMS measurements, respectively, LaMoury et al. (2021) and Goncharov et al. (2020) concluded that jets behind the quasi-parallel shock can propagate deeper into the magnetosheath. Raptis et al. (2020a), however, reported that jets behind the quasi-perpendicular shock appeared to be closer to the magnetopause on average compared to the quasi-parallel jets. They noted, however, that this effect could partly be explained by the mean solar wind conditions during quasi-perpendicular jet detection and the model used to infer the position. In contrast to this, Goncharov et al. (2020) reported jets only up to 2.5 R_*E*_ downstream of the quasi-perpendicular shock. Investigating the region close to the quasi-perpendicular shock, Vuorinen et al. (2023a) reported jet-like enhancements within the shock transition region, which were attributed the quasi-perpendicular shock dynamics. Recently, Pöppelwerth et al. (2024b) presented a list of jets detected by the Cluster spacecraft using three different detection criteria. The jet occurrence appeared to be more concentrated in the middle of the magnetosheath compared to previous findings using THEMIS and MMS. Different jet criteria affected the spatial distribution: jets detected via the Plaschke et al. (2013) criterion are more frequently observed closer to the bow shock than jets based on the criteria by Archer and Horbury (2013) and Koller et al. (2022).

With respect to numerical simulations, Palmroth et al. (2018) used the global hybrid-Vlasov code Vlasiator to investigate a magnetosheath jet behind the quasi-parallel shock which fulfilled the three different observation-based criteria proposed by Karlsson et al. (2012), Plaschke et al. (2013) and Archer and Horbury (2013), respectively. Their identified jet region was continuous, starting from the shock and moving towards the magnetopause. They found that using the Plaschke et al. (2013) criterion led to jet detection being mostly fulfilled near the bow shock. The Karlsson et al. (2012) criterion was also mostly fulfilled near the bow shock and rarely close to the magnetopause. Jets observed closer to the magnetopause were better identified using the Archer and Horbury (2013) criterion as dynamic pressure decreases as a function of distance from the bow shock. Suni et al. (2021), also using Vlasiator, analysed the connection between foreshock compressive structures (FCSs) impacting the bow shock and jet formation. FCSs were defined as localised increases in both dynamic pressure and magnetic field strength in the ion foreshock. Because of the setup using an almost radial IMF, their results focus on the quasi-parallel magnetosheath region. They analysed the disappearance rate of jets depending on the distance to the bow shock. Jets related with FCSs propagated around twice as far into the magnetosheath compared to non-FCS-jets. Using a set of local 2D hybrid simulations with a variety of different $\theta _{Bn}$ and Alfvénic Mach numbers Tinoco-Arenas et al. (2022) also reported that number of jets decrease with the distance from the shock for all the observation-based criteria (see Sect. 2.1.1). The events that were found at largest distances from the shocks were those corresponding to high-speed jets as defined by Plaschke et al. (2013) and transient flux enhancements as defined by Němeček et al. (1998).

#### Jet Occurrence in Other Environments

Shocks are a ubiquitous phenomena observed across the interplanetary medium with a variety of scales, Mach numbers and curvature radii. Investigating jets in other downstream shock environments, such as those generated by CMEs and SIRs or other planetary bow shocks, could provide deeper understanding of the universality of jet formation, shock physics, and the scaling of dimensionless parameters.

Karlsson et al. (2016) searched for jets in Mercury’s environment using data from the MErcury Surface, Space ENvironment, GEochemistry, and Ranging (MESSENGER) mission (Solomon et al. 2001). However, the mission was not designed for this type of study so the instrumentation was incompatible with the objective of identifying jets. The instrumental payload could not provide the required measurements of plasma velocity and density. Instead, using solely magnetic field data, plasmoids related to magnetic holes and flux transfer events were identified. This study is discussed in further detail in the review paper by Plaschke et al. (2018).

**Fig. 6:**
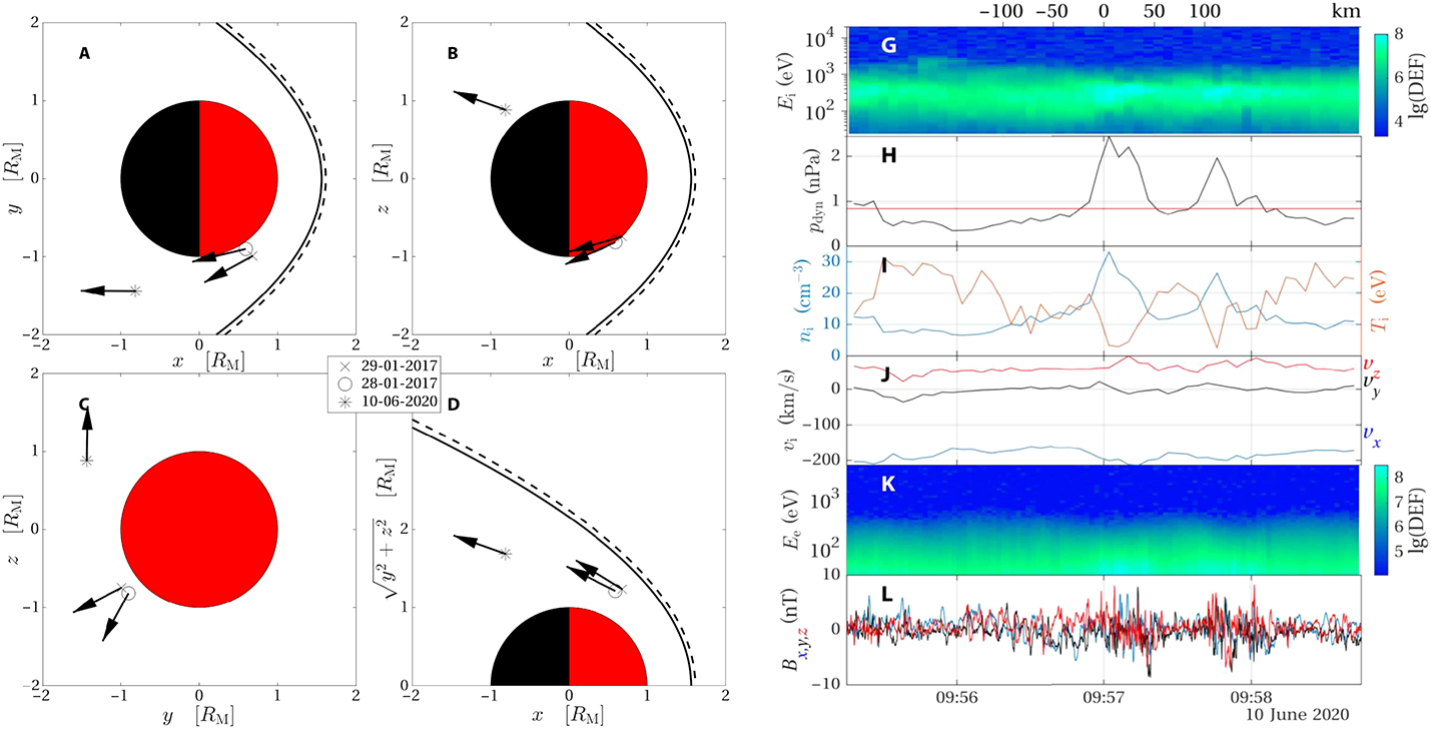
Jet detection in the magnetosheath of Mars. Panels A, B, C, and D show the position of the MAVEN spacecraft during the detection of three jets in Mars Solar Orbital coordinates. The right hand plot shows measurements of one of the detected jets: Ion differential energy flux G), ion dynamic pressure H), ion density and temperature I), ion velocity components J), electron differential energy flux K), and magnetic field components L). Images reproduced with permission from Gunell et al. (2023), copyright by the author(s)

Recently, jets were confirmed in another space environment than Earth’s. Gunell et al. (2023) found evidence of jets occurring in the Martian magnetosheath using data from the Mars Atmosphere and Volatile EvolutioN (MAVEN) mission (Jakosky et al. 2015). As the induced magnetosphere of Mars is significantly smaller than Earth’s intrinsic magnetosphere, the criterion of jet detection had to be modified. The Archer and Horbury (2013) criterion of the dynamic pressure being at least twice of the average dynamic pressure over a 20-minute interval for a jet was adjusted to a 10-minute interval instead. Figure 6 shows both the position of the detected jets and an example of the plasma measurements for one of the events.

Three detected jets were studied, selected over a wide spatial extent. It was found that two were related to an increase in both density and absolute value of the velocity, while the third showed only a density increase. The lack of speed enhancement on the third jet was explained by its location deeper into the magnetosphere. This is comparable with Earth-based jets, as they are known to slow down with time spent in the magnetosheath (Goncharov et al. 2020). Furthermore, all three cases exhibited a higher degree of magnetic field fluctuation than the background, indicating that the jets promoted wave excitement.

Shortly after the discovery of jets at Mars, Zhou et al. (2024) showed for the first time evidence of jets being present in the Jovian magnetosheath. Using data from the Voyager 2 mission (Kohlhase and Penzo 1977), one sunward and two anti-sunward jets could be identified as the spacecraft travelled in the subsolar magnetosheath of Jupiter. It was found that the magnetic field of the sunward jet experienced a decrease in magnitude and rotation in its direction in relation to the ambient plasma. It was possible to rule out HFAs as a cause for this jet due to the increase of density. The two anti-sunward jets, on the other hand, gave indications that an HFA could have caused their formation as pile-ups at its edges. This was motivated by a decrease of density, increase of temperature and rotation of magnetic field during the short period between the two jets. Lastly, when comparing the Jovian jets with jets from Mars and Earth, Zhou et al. (2024) could establish a scalable relationship between jets’ and their environmental system’s size.

Hietala et al. (2024) reported the first-ever observation of jet-like structures in non-planetary shock environments. They used a modified jet selection criteria based on the compression ratio suitable for low Mach number shocks. At high Mach numbers the criterion reduces to the (Plaschke et al. 2013) jet criterion. They reported jet candidates downstream of three interplanetary shocks measured by the Wind spacecraft (Wilson et al. 2021). All three events show the jet-like structures in the quasi-parallel or oblique region. One shock shows a high Mach number (similar to Earth’s bow shock), while the other two shocks show very low mach numbers and low plasma beta. They discuss that for two of their presented events the creation of jets via shock reformation and shock non-stationarity are plausible, while the third shock indicates a jet formation due to a magnetic field discontinuity.

### Formation Mechanisms

The origins of most magnetosheath jets have been associated with various properties of the Earth’s bow shock, including quasi-parallel shock conditions and the existence of a foreshock. Specifically, jets can form as a result of shock non-stationarity, or by the interaction of upstream transients with the shock (Plaschke et al. 2018).

#### Shock and Foreshock

**Fig. 7:**
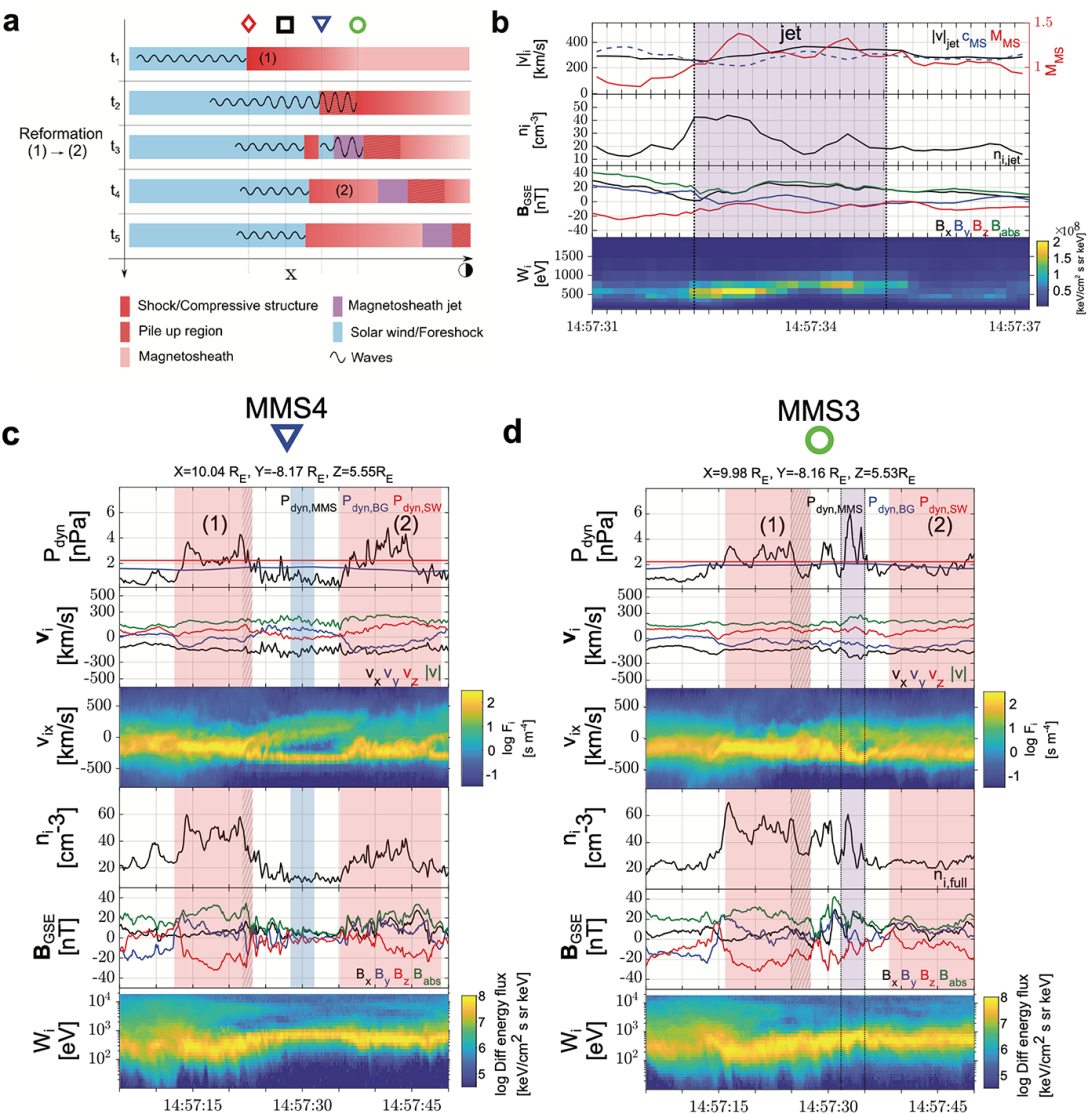
Jet formation mechanism due to shock reformation cycle. (**a**) shows a schematic of the reformation cycle as seen by the MMS spacecraft in string-of-pearls configuration. MMS2 and MMS1 (red diamond and black square) observed primarily upstream solar wind measurements (not shown here), while MMS4 actively observed the reformation cycle over temporal scales of less than a minute. MMS3, downstream of the shock, observed the solar wind embedded with its upstream wavefield forming a high-speed jet. In panel (**b**) a zoomed-in plot of the jet observation is shown where partial moments highlight how the jet maintained its supermagnetosonic property from its solar wind origin, surrounded by relative density enhancements caused by the embedded wavefield. On the right, (**c**) shows MMS4 and (**d**) MMS3 observations. Panels, [top - bottom]: Dynamic pressure enhancement with background values from surrounding plasma and solar wind (OMNI) values, ion velocity components, 1D VDF in X GSE , ion density, magnetic field components and differential ion energy spectra. Images adatpted from Raptis et al. (2022b), copyright by the author(s)

Quasi-parallel shock reformation has been associated with jet observations. Related to a kinetic formation of jets, Raptis et al. (2022b) showed in-situ evidence of a jet forming due to the quasi-parallel shock reformation cycles and the evolution of upstream waves. They used MMS in a string-of-pearls configuration at the bow shock to track individual structures during the shock reformation process. Figure 7 shows an illustration of the observed event, indicating that solar wind parcels in-between compressive foreshock structures retained their solar wind-like properties and formed a jet as they became part of the magnetosheath.

The formation of jets has also long been associated with shock ripples (Hietala et al. 2009; Hietala and Plaschke 2013; Hao et al. 2016). However, while recent studies have supported this picture, they have also indicated that other jet generation mechanisms are at play. The rippled shock mechanism has been supported by statistical studies (Raptis et al. 2020a) that showed temperature and velocity changes to be anti-correlated. That indicates, as the mechanism suggests, a less heated solar wind-like plasma being associated with jet observations. Furthermore, recent hybrid simulation results show jets originating downstream of a rippled quasi-parallel shockfront: In Preisser et al. (2020) a jet is formed downstream of a supercritical ($M_{A}=7$) shock with $\theta _{Bn}=15^{\circ}$ following the mechanism proposed by Hietala et al. (2009). Ren et al. (2023) performed 2D hybrid simulations of quasi-parallel shocks. In the study they found that the interaction between upstream compressive structures and the shock front produce shock rippling and jets just downstream of the rippled shock dents. In their $\theta _{Bn}=0^{\circ}$ simulation the generated jets can sustain multiple reformation cycles. The authors conclude that these jets are not a direct result of the shock reformation process as described by Raptis et al. (2022b).

**Fig. 8:**
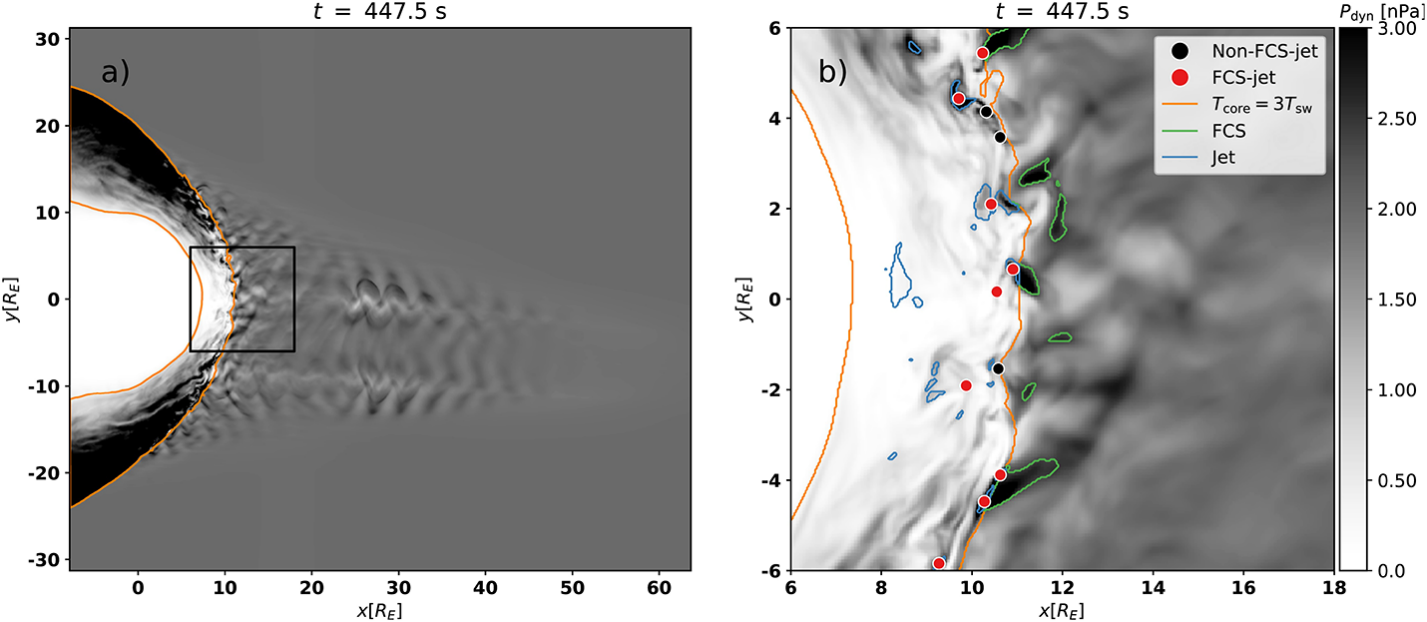
Jets formed after impact of foreshock compressional structures in 2D hybrid simulations. Panel a) shows the global domain, panel b) a zoom-in on the dayside bow shock environment. FCS (green) impacting the bow shock cause jets (red dots) downstream of the impact area. Input parameter for this simulation run: total magnetic field: 5 nT, cone angle: 5^∘^, density: 3.3 cm^−3^, velocity (sunward): −600 km/s, Alfvén Mach number: 10. Image reproduced with permission from Suni et al. (2021), copyright by the author(s)

The role of compressive structures such as SLAMS in the formation of jets has been investigated and discussed in recent studies. SLAMS themselves are magnetic pulsations with a significant increase in magnetic field strength with a typical duration of 10 s. Using the global hybrid-Vlasov code Vlasiator, Palmroth et al. (2018) investigated a magnetosheath jet which formed behind the quasi-parallel bow shock. The studied jet was formed by the interaction of a high-dynamic-pressure structure (with the characteristics of SLAMS) with the bow shock. This mechanism was also observed in the local 2D hybrid-PIC simulation by Hao et al. (2016). According to Palmroth et al. (2018), SLAMS can pass the bow shock with little braking and propagate deep into the magnetosheath thanks to a preexisting dent in the bow shock. This is in agreement with Karlsson et al. (2015) who suggested that SLAMS can cross the bow shock maintaining higher pressure if there is a corrugation at the bow shock upon which the SLAMS are incident. Suni et al. (2021) further studied FCSs impacting the bow shock and their relation to jet formation using 2D global hybrid-Vlasov simulations. They found that 75% of the forming jets were in fact FCSs, which were continually transformed into jets at the bow shock. Figure 8 shows results from a simulation run where jets formed after the impact of FCSs at the bow shock. In a follow-up study, Suni et al. (2023) found that the non-FCS jets in Suni et al. (2021) could be classified as flankward or antisunward, depending on their direction of propagation. They showed that antisunward jets had the same properties and origin as FCS jets in Suni et al. (2021), meaning that $86\%$ of jets that formed at the bow shock under steady solar wind conditions and quasi-radial IMF in all the simulation runs were associated with FCSs. Furthermore, Raptis et al. (2022b) showed through MMS observations that SLAMS downstream of the shock may appear as jets (or “plasmoids” if following the definitions of Karlsson et al. 2015) due to their relatively higher density. In addition, Xirogiannopoulou et al. (2024) found that compressive foreshock subsolar structures (SLAMS, plasmoids, and mixed structures) have an increased appearance rate with increasing pristine solar wind velocity, similar to magnetosheath jets. However, foreshock SLAMS were detected more frequently in denser regions in contrast to magnetosheath jets (LaMoury et al. 2021). The statistical analysis by Xirogiannopoulou et al. (2024) showed that density-enhanced structures (primarily plasmoids, but also mixed structures) had dynamic pressure enhancements, rather than the magnetic enhanced structures (SLAMS). By combining the previous studies by Suni et al. (2021), Raptis (2022), and the comparative pristine solar wind results of all the previous magnetosheath jet studies, Xirogiannopoulou et al. (2024) proposed that structures with increased densities are the most probable sources of magnetosheath jets.

Regardless of the origin of the non-stationarity of the shock (i.e., reformation/ripples), the root of such effects is the presence of the ion foreshock and its transient phenomena. Therefore, the above research along with several others have effectively associated the formation of jets with foreshock activity.

In quasi-perpendicular shocks, due to the absence of an extended ion foreshock, the building blocks of large scale non-stationarity are absent and jets are typically not associated to bow shock dynamics. However, quasi-perpendicular ripples (not to be confused with quasi-parallel ripples) are typically present (Johlander et al. 2016) and it has been hypothesised that they could be responsible for generating small-scale jets in the quasi-perpendicular magnetosheath (Raptis et al. 2020a). Furthermore, recently it has been shown that ion gyromotion at the quasi-perpendicular shock transition layer can lead to jet-like observations (Vuorinen et al. 2023a).

Sibeck et al. (2021) showed jet-like observations when foreshock compressional boundaries (FCBs) get transmitted downstream. They used a global hybrid code to predict the transmission of enhanced flow into the magnetosheath. Their simulation showed results for a quasi-stationary FCB as well as a travelling FCB coming from a foreshock cavity (also called travelling foreshock). Upstream and downstream observations of a foreshock cavity by THEMIS confirmed their results. FCBs exhibit density and magnetic field enhancements upstream, while their transmitted counterparts in the magnetosheath show enhanced flow speeds and depressed temperatures in addition to the density and magnetic field enhancements. Their dimensions along the flow axis appeared to be greater than the dimensions transverse to it. These properties match with the commonly understood properties of jets in the magnetosheath.

#### Other Formation Mechanisms

While latest research on jet formation has primarily focused on the connection with the shock and foreshock region, some studies have examined formation mechanisms that are not directly connected to shock physics in steady solar wind conditions.

Archer et al. (2012) proposed a jet generation mechanism associated with the rotation of the magnetic field. These jets thus appear at the local interface from the quasi-parallel to the quasi-perpendicular region caused by solar wind discontinuities. One jet analysed by Blanco-Cano et al. (2020) was attributed to this mechanism.

Kajdič et al. (2021) analysed the origin of jets in the magnetosheath region downstream of the quasi-perpendicular shock. They showed four different event types with characteristic signatures for jets. One jet type in the quasi-perpendicular magnetosheath was caused by magnetic flux tubes connected to the quasi-parallel shock. The plasma properties inside these magnetic flux tubes showed similar properties to the quasi-parallel magnetosheath. Either the rims or the inside of the flux tubes may have produced jet-like signatures, surrounded by quasi-perpendicular plasma. Consequently, these jets would have been classified as “encapsulated jets” by Raptis et al. (2020a). Kajdič et al. (2021) noted that these flux tubes were equivalent to foreshock cavities and travelling foreshocks in the upstream solar wind plasma.

Kajdič et al. (2021) also discussed how reconnection exhausts in the magnetosheath may cause jet-like signatures in the quasi-perpendicular magnetosheath. Reconnection exhausts are Alfvénic flows confined to regions with a large change in magnetic field direction. The authors noted that the quasi-perpendicular magnetosheath can contain ion jets embedded in reconnection exhausts because the plasma exhibits a low level of turbulence, while the higher degree of turbulence seen in the quasi-parallel magnetosheath explains the lack of ion jets found there (Phan et al. 2018). Blanco-Cano et al. (2020) examined two jets in MMS data that were by-products of magnetic reconnection at the magnetopause. These events showed primarily southward-directed motion and two different ion populations. One ion population was a dense, unaccelerated population from the magnetosheath and the other one was a less dense, accelerated population from the magnetosphere. The event also showed indications of two electron populations with different pitch angle distributions. However, not all of the characteristic reconnection exhaust signatures were observed in the plasma data, perhaps due to the distance of the observations from the corresponding X-line. For example, the event lacked large IMF shear angles and the relation between $\mathbf{B}$ and $\mathbf{V}$ at both edges of the event.

**Fig. 9:**
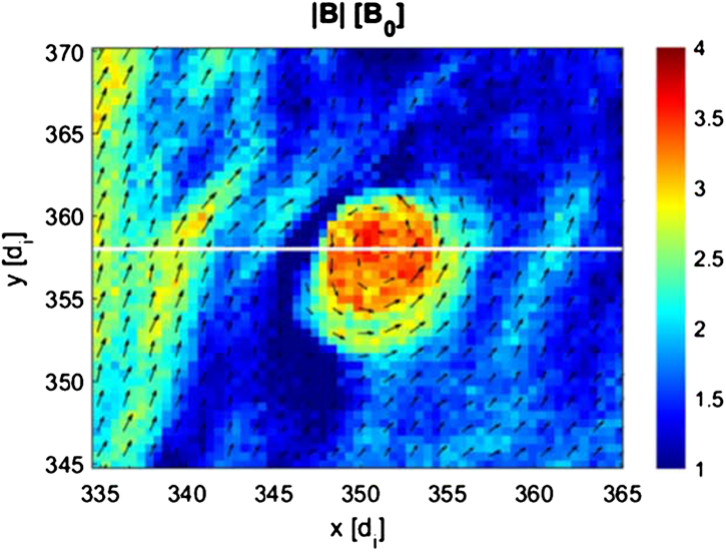
Plasmoid with self-consistent structure formed after reconnection behind a quasi-parallel shock in 2D local hybrid simulations. The colour scale indicates the magnetic field amplitude in nT and the black arrows show the local magnetic field vectors. Image reproduced with permission from Preisser et al. (2020), copyright by the author(s)

Using a 2D local hybrid simulation, Preisser et al. (2020) reported a paramagnetic embedded plasmoid (defined as an increase in both density and magnetic field strength, similar to Karlsson et al. 2015) formed due to magnetic reconnection at a quasi-parallel shock ($\theta _{Bn} = 15^{\circ}$). The authors suggested that SLAMS were piled up behind the shock due to local reformation processes. Eventually, SLAMS became part of the downstream region where magnetic reconnection occurred due to the change in magnetic field orientation. This process led to the formation of a plasmoid, a magnetically isolated structure with inhibited diffusion during its transport downstream (see Fig. 9).

Omelchenko et al. (2021) studied the generation of jets in Earth’s dayside magnetosphere using the hybrid-PIC model HYPERS to set up simulations with southward and northward quasi-radial IMF ($\theta _{Bn} =\pm 10^{ \circ}$). In their simulations, jets naturally formed as high-density solar wind plasma structures. These structures were associated with magnetic field filaments dynamically emerging during the turbulent motion of magnetosheath plasma. Crucially, jets did not appear directly behind the rippled shock front as proposed by Hietala et al. (2009, 2012). As the origin of these jets appeared to be closely tied to the turbulent dynamics of the magnetic field, Omelchenko et al. (2021) proposed a “magnetokinetic” mechanism for their origin. In this system, solar wind plasma interacts with magnetosheath turbulence, driving magnetic field perturbations with dynamic pressure enhancements. The properties are generally consistent with jet observations.

Suni et al. (2023) found that flankward jets in the magnetosheath (14% of all investigated jets) differed from jets related with FCSs (Suni et al. 2021) using global 2D hybrid-Vlasov simulations (Vlasiator). The identified flankward jets were not associated with foreshock structures and showed characteristics of quasi-perpendicular magnetosheath plasma. These included high temperature anisotropy, enhanced magnetic field, and smaller changes in velocity, which are typical for quasi-perpendicular magnetosheath jets (Raptis et al. 2020a). They suggested that the formation of flankward jets could be related with local changes from a quasi-parallel to quasi-perpendicular bow shock. Suni et al. (2023) argued that the formation of those flankward jets could be connected to foreshock ULF wave activity at the oblique shock region.

Another jet-type discussed by Kajdič et al. (2021) was connected to the upstream edge of a non-reconnecting current sheet. The authors proposed that the magnetic field gradient or the curvature of the current sheet may produced ion drifts, resulting in increased ion velocity and thus dynamic pressure. The examined event showed similarities to reconnection exhausts, however the structure did not show the magnetic field and velocity correlations required to classify it as such.

Mirror modes (see Tsurutani et al. 2011, and references therein) may also coexist with jet-like signatures in the quasi-perpendicular magnetosheath (Blanco-Cano et al. 2020; Kajdič et al. 2021; Blanco-Cano et al. 2023). Mirror modes are characterised as compressive magnetic field fluctuations that are anticorrelated with the plasma density. These modes usually take the form of magnetic field dips or peaks with respect to the surrounding plasma. Kajdič et al. (2021) showed an example of a dynamic pressure enhancement that was observed during a magnetic field dip and during a time when the mirror mode instability parameter was fulfilled. The interaction of mirror modes with jets and any resulting effects on jet properties and microstructure ought to be investigated in more detail through an extended survey of coexisting structures.

**Fig. 10:**
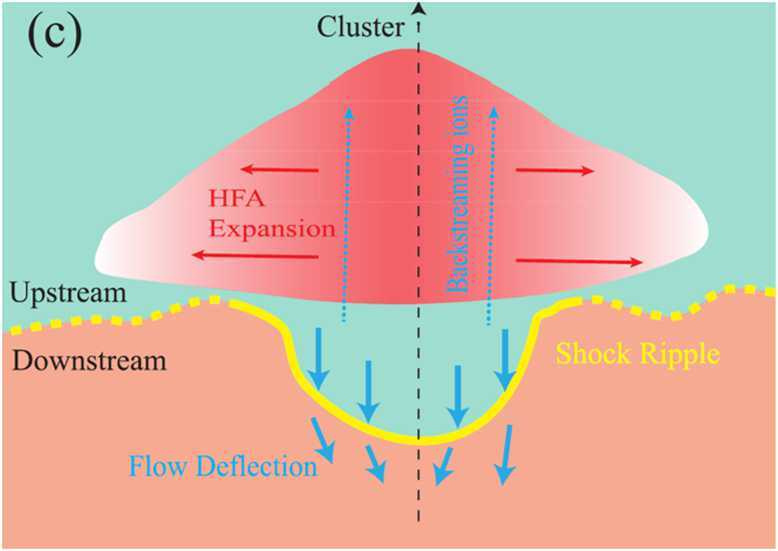
Proposed formation mechanisms of jets caused by both an HFA and associated ripples in the bow shock. Image reproduced with permission from Zhou et al. (2023), copyright by the author(s)

Zhou et al. (2023) proposed that the interaction of upstream discontinuities with a quasi-perpendicular shock could produce both an HFA and a downstream jet by causing large-scale undulations at the shock. This would combine the ripple mechanism (Hietala et al. 2009) with the shock-discontinuity interaction mechanism (Archer et al. 2012). Figure 10 shows a schematic for this formation process involving HFAs and ripples.

Recent work by Osmane and Raptis (2024) proposed that magnetosheath jets can also form due to local kinetic instabilities near the bow shock transition. Specifically, firehose-unstable fluctuations and compressive heating within collisionless plasma environments were shown to theoretically amplify kinetic energy density. Preliminary MMS observations downstream of quasi-parallel bow shocks indicate that a subset of jets exhibit firehose-unstable plasma conditions, providing support to this theoretical framework.

### Discussion

Several studies have focused on examining the statistical occurrence and spatial distribution of jets within the magnetosheath in relation to upstream solar wind conditions. Jet occurrence in the dayside magnetosheath is primarily increased by the existence of the quasi-parallel bow shock and consequently the foreshock near the subsolar region. It has been shown that other solar wind parameters influence the jet occurrence as well, albeit to a lesser degree. In terms of spatial occurrence, magnetosheath jets are more often detected close to the bow shock, indicating that most form there and dissipate over time while moving through the magnetosheath. Therefore, studies on jet formation might benefit of selection criteria restricted to jets observed close to the bow shock to remove the effects of propagation through the magnetosheath (LaMoury et al. 2021). The majority of jet formation is clearly related to the quasi-parallel bow shock. However, the exact dominant generation process (like shock rippling, foreshock structures, shock reformation or a combination of several processes) is still open for discussion due to the lack of statistical studies exploring these formation mechanisms.

Jet occurrence investigations using in-situ measurements are heavily limited (and thus possibly biased) by orbits of the spacecraft. Vuorinen et al. (2023b) showed that the apogee of spacecraft orbits can heavily influence long term jet occurrence statistics, because more jets are detected closer to the bow shock. Recent years have shown an increase in statistical jet research focused on the dayside magnetosheath and subsolar region only (e.g. Vuorinen et al. 2019; LaMoury et al. 2021; Koller et al. 2022). There have only been a few studies on jets and their formation in the flanks of the magnetosheath (e.g. Raptis et al. 2020a), where we might see different occurrence, behaviour and evolution.

Future missions with larger spacecraft distances allowing simultaneous measurements in the foreshock, at the bow shock, and deeper within the magnetosheath could be used to further study the formation on a larger spatial scale. Rapid improvements in simulations, such as the development of 3D ion kinetic simulations with adaptive resolution capturing foreshock, bows shock, and magnetosheath (i.e. Ganse et al. 2023), can provide more insights into the formation of jets and where they occur. Simulations driven by solar wind observations (including changing parameters and turbulence) can shed more light on jet formation at the bow shock in the future.

## Jet Properties and Evolution

In the previous section, it was discussed that jet occurrence is primarily associated with the quasi-parallel bow shock and the foreshock, with jets forming at the bow shock. In order to understand how jets interact with and exchange energy with the surrounding plasma, as well as which jets have the potential to impact the magnetopause, knowledge about the properties and evolution of jets is required. In recent years, these have been studied using both case and statistical studies of spacecraft observations, as well as using hybrid-kinetic simulations.

### Bulk Properties and Internal Structure

An assessment on the internal structure of jets, comprising the distribution of velocities, bulk and electromagnetic field properties, and physical morphology, is key to understanding how they interact with their environment. We will discuss each of these elements in turn over the course of this section.

#### Velocity Distribution Function Properties

A few studies have used spacecraft data to probe the velocity distributions of ions inside magnetosheath jets. Karlsson et al. (2018) used observations from the MMS spacecraft constellation to study six different jets during two time periods in 2015, Blanco-Cano et al. (2020) studied three magnetosheath jets observed by MMS during a 45-minute interval with southward magnetic field ($B_{z}<0\text{ nT}$) in the dayside magnetosheath, and Raptis et al. (2022a) investigated a magnetosheath jet downstream of the quasi-parallel bow shock in a strongly turbulent magnetosheath region using MMS data.

**Fig. 11:**
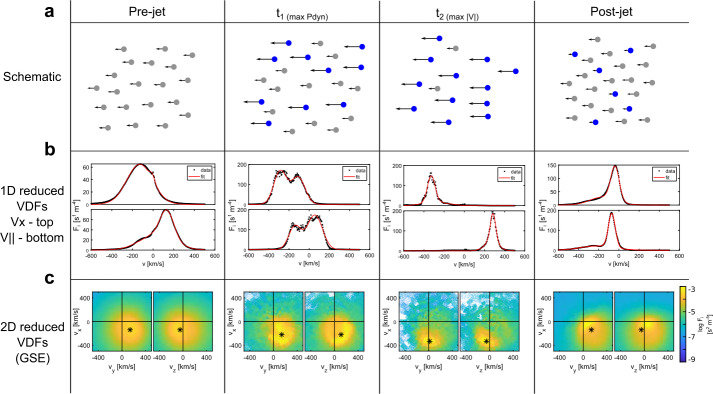
Evolution of the ion velocity distribution function around the time of a jet observation. The pre-jet distribution is averaged from 50 measurements before the jet, while the post-jet distribution is averaged from 40 observations after the jet. Panel (a) shows a schematic of the evolution of individual particle velocities, (b) shows VDFs reduced to 1D in the $v_{x}$ and $v_{\parallel}$ directions, and (c) shows 2D reduced VDFs with asterisks marking the bulk velocity. Image reproduced with permission from Raptis et al. (2022a), copyright by the author(s)

Karlsson et al. (2018) found two jets that straddled the boundary between the quasi-parallel and quasi-perpendicular magnetosheath. The jets consisted of different plasma populations, cooler than the surrounding ion populations, in different parts of the jets. The leading parts exhibited isotropic, quasi-parallel magnetosheath-like temperature, while the trailing parts exhibited temperature anisotropy $(T_{\perp}/T_{\parallel }> 1)$ reminiscent of the quasi-perpendicular magnetosheath. These jets exhibited correlation between density, velocity, dynamic pressure, and magnetic field. Blanco-Cano et al. (2020) also found two velocity-driven jets with temperature anisotropy, but in their case $T_{\perp}/T_{\parallel }< 1$. The authors also observed secondary field-aligned beams, suggesting an association with dayside reconnection. The jet studied by Raptis et al. (2022a) was also found to consist of multiple populations: A cold and fast jet population, and a hot but slow background population (see Fig. 11). The jet population had a solar wind-like velocity and slightly enhanced density.

The remaining jets studied by Karlsson et al. (2018) consisted of ion populations similar to, but cooler than, the surrounding magnetosheath. Three of the jets exhibited isotropic temperature, while one exhibited anisotropy $T_{\perp}/T_{\parallel }> 1$. The remaining jet in Blanco-Cano et al. (2020) also consisted of an isotropic ion population, but was associated with a magnetic field rotation. This jet was density-driven.

#### Bulk and Magnetic Field Properties

As more and more high-quality spacecraft data from, and simulation data of, the magnetosheath has become available, more statistical studies of magnetosheath jet properties have been conducted. Goncharov et al. (2020) used MMS data and OMNI solar wind data from 2015-2017 to analyse 1400 jets and fast plasmoids – structures of enhanced $x$-directional dynamic pressure and velocity – in the magnetosheath during a wide range of magnetosheath and solar wind conditions. Raptis et al. (2020a) analysed ∼8500 jets observed by MMS. Echim et al. (2023) studied 960 jets observed by the Cluster 3 spacecraft in 2007 and 2008. Tinoco-Arenas et al. (2022) conducted a statistical study based on a set of local 2D hybrid simulations using different IMF angles and Alfvén Mach numbers at the shock.

Different studies have found contrasting results regarding the density inside jets. Often, the disparities can be attributed to the different regions where the jets were found. Jets located in the quasi-parallel magnetosheath often showed density increases (Raptis et al. 2020a; Echim et al. 2023), whereas jets behind the quasi-perpendicular shock showed both increases (Blanco-Cano et al. 2020) and hardly any increase in density (Raptis et al. 2020a). Jets between these regions, in Raptis et al. (2020a) referred to as ‘boundary jets’ and ‘encapsulated’ jets, also exhibited an increased density.

Several studies reported that the magnetic field magnitude increases inside jets. Karlsson et al. (2018) observed a correlation between magnetic field strength and velocity for jets associated with an upstream magnetic field discontinuity and for jets in the quasi-parallel magnetosheath with a simpler substructure in velocity. In Raptis et al. (2020a), jets in the quasi-parallel magnetosheath also show a positive correlation between density increase and magnetic field increase. Goncharov et al. (2020) reported that the majority of jets and plasmoids show an increased magnetic field strength; however, the magnetic fields in jets and plasmoids are not identical and could indicate different formation mechanisms.

As already reported in the review of Plaschke et al. (2018), the temperature inside the majority of the jets is lower than in the surrounding magnetosheath. These findings were confirmed in Karlsson et al. (2018), Raptis et al. (2020a,b). However, Echim et al. (2023) observed a positive correlation between perpendicular temperature and magnetic field intensity, which they speculated could be due to an adiabatic braking process.

The majority of these studies reported that jets are usually associated with earthward velocity enhancements Goncharov et al. (2020), Raptis et al. (2020a), Karlsson et al. (2018), Tinoco-Arenas et al. (2022), which often naturally follows from the criteria used to identify the jets. However, Raptis et al. (2020a) reported a few jets with either very small earthward or even sunward velocities. They proposed that these jets could have been formed through plasma reflection at the magnetopause.

#### Morphology

A few recent studies have investigated the size and shape of magnetosheath jets. Goncharov et al. (2020) determined the size of jets detected by MMS assuming a cylindrical jet geometry. They estimated the parallel size by integrating the ion velocity over the jet duration and the perpendicular size (or thickness) by estimating the flow speed normal to the jet flow direction. They reported that the typical jet size is several thousands of kilometres, increasing with distance from the bow shock. Furthermore, jets are almost twice as large in the direction parallel to the jet’s plasma flow than perpendicular to it.

Plaschke et al. (2020a) proposed a new model for the unbiased size distribution for jets. The model accounts for the observational bias of jets with larger cross-sectional area having a higher probability to be observed by a spacecraft. For our discussion, we briefly review findings by Plaschke et al. (2016) which were already discussed by Plaschke et al. (2018). Plaschke et al. (2016) utilized two-spacecraft THEMIS observations and calculated how likely it is that two spacecraft at a given separation distance observe the same jet when the jet is propagating nearly perpendicular to the separation vector between the spacecraft. They compared the observation rates to those expected for jets with a given size distribution. Assuming that jets have circular cross-sections, they ultimately estimated that the distribution of diameters $D_{\perp}$ of the observed jets followed an exponential form. However, Plaschke et al. (2020a) noted that, having applied the correction factor $1/D^{2}_{\perp}$, the obtained probability density function could not be appropriately normalised, leading to an overestimation of the observation rates of smallest-scale jets. Therefore, they instead proposed a new model for the unbiased size distribution of jets, a log-normal distribution. The parallel sizes of the jets were estimated by integrating the ion velocity over the jet duration, and they were similarly modelled with a log-normal distribution. Using the unbiased distributions, the authors estimated a median perpendicular scale size of 0.12$\,R_{\mathrm{E}}$ and a median parallel scale size of 0.15$\,R_{\mathrm{E}}$. These true scale sizes are significantly smaller than the median observed scale sizes: 0.89$\,R_{ \mathrm{E}}$ and 0.68$\,R_{\mathrm{E}}$, respectively.

Using multispacecraft observations from the THEMIS mission, Pöppelwerth et al. (2024a) developed a method for estimating the sizes of jets perpendicular to the direction of propagation and applied the method to a single jet. They found that the perpendicular size increased from the leading part of the jet toward the center and decreased from the center toward the trailing part, with the maximum size being $1.2\,R_{\mathrm{E}}$. The maximum size is co-located with the location of maximum dynamic pressure (6 nPa), which also decreases away from the center toward the leading and trailing parts.

A comparison of the results of Goncharov et al. (2020) and Plaschke et al. (2020a) shows that in both studies the jets are more elongated in the direction parallel to the flow. The median observed scale sizes are also similar in both studies: $0.8-0.9\,R_{\mathrm{E}}$ (Plaschke et al. 2020a) compared to a few thousand kilometres (Goncharov et al. 2020). This scale size also agree with the $1.2\,R_{\mathrm{E}}$ estimated by Pöppelwerth et al. (2024a). However, this observed scale size is biased and the unbiased scale size is smaller, on the order of $0.1\,R_{\mathrm{E}}$. This highlights the importance to account for possible observational biases when investigating the scale sizes of magnetosheath jets.

In two similar studies of magnetosheath jets using two different 3D hybrid-PIC simulations, Fatemi et al. (2024) and Ren et al. (2024a) independently found that the shapes of magnetosheath jets during quasi-radial IMF conditions are very different from the pancake-, cylinder-, or sphere-like shapes inferred in the past from spacecraft observations and 2D simulations. Both studies found that in the plane perpendicular to the solar wind flow, magnetosheath jets appear as thin, interconnected filamentary structures of higher dynamic pressure surrounding larger, round regions of lower dynamic pressure. Ren et al. (2024a) call this shape “honeycomb-like”.

### Jet Evolution

The study of the evolution of magnetosheath jets concerns the ways in which their aforementioned properties change as a function of time and location in the magnetosheath. Tracking the evolution of a single jet over time with spacecraft observations is a challenging task as it requires fortuitous positioning of multiple spacecraft, and sufficient distance between them due to the large sizes of jets, as well as a method for confirming that the jets observed by all spacecraft are the same. Most studies of jet evolution so far have therefore relied on numerical simulations, although a few statistical spacecraft observation studies also offer insight into the magnetosheath penetration depth, size and shape, and propagation of jets forming under different solar wind and IMF conditions.

#### Evolution of Plasma and Magnetic Field Properties

**Fig. 12:**
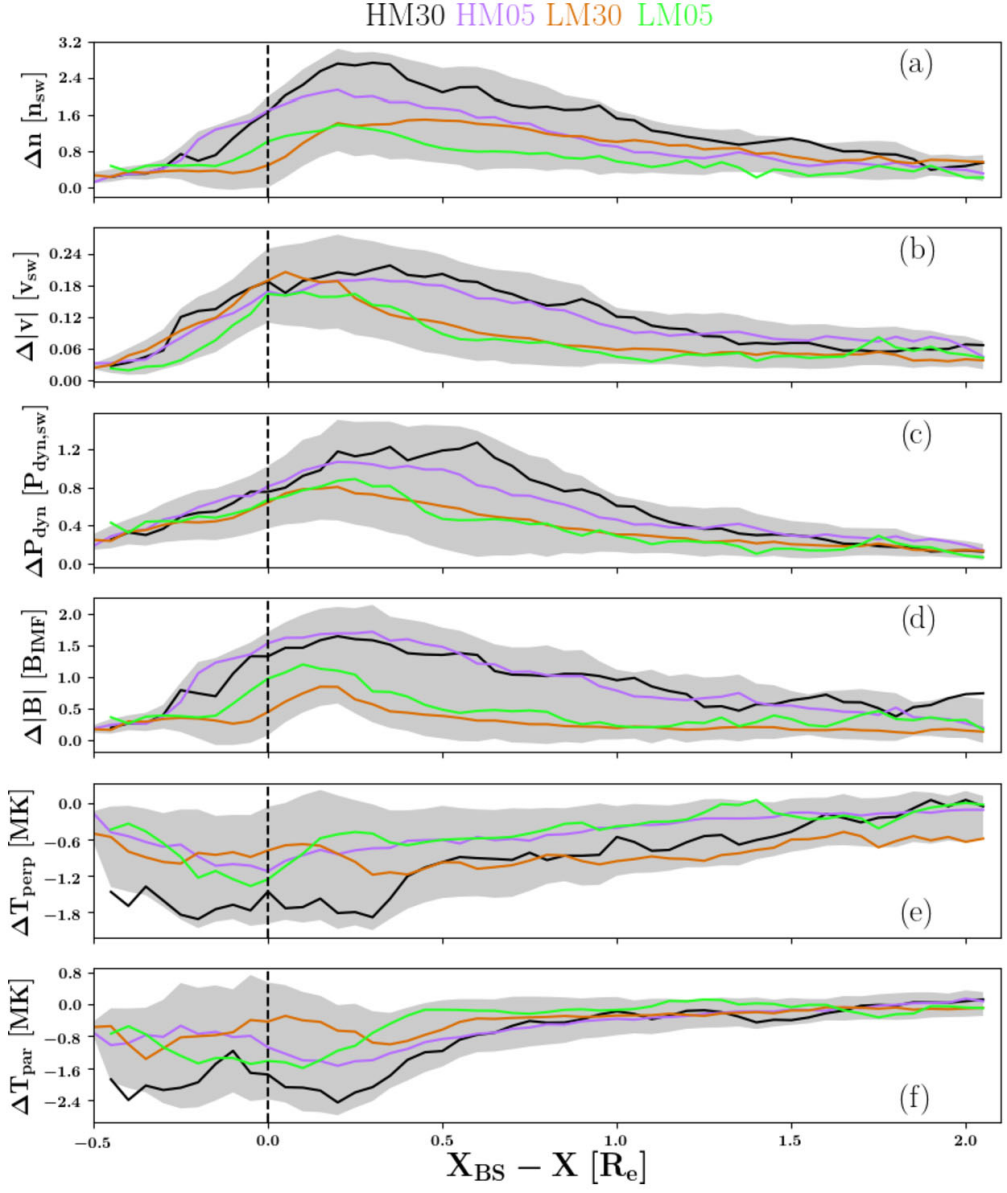
Superposed epoch study of the differences ($\Delta $) between jet and ambient magnetosheath in (a) density, (b) velocity, (c) dynamic pressure, (d) magnetic field intensity, (e) perpendicular temperature, and (f) parallel temperature, as a function of distance from the bow shock for simulated jets. The temperature differences are given in units of MK, while for the other variables the differences are normalised to solar wind values. The results are shown for 4 different simulation runs, plotted as different colours: The HM (LM) runs have an IMF strength of 5 (10) nT, while the 05 (30) runs have an IMF cone angle of 5^∘^ (30^∘^). The grey filled-in area shows the standard deviation from the average of all jets. Image reproduced with permission from Palmroth et al. (2021), copyright by the author(s)

As jets propagate in the magnetosheath, they interact and change the ambient plasma and magnetic field. Palmroth et al. (2018) studied a single jet forming at the bow shock in a 2D simulation run of the global hybrid-Vlasov model Vlasiator and found that the initial dynamic pressure enhancement at the bow shock was associated mainly with a sharp increase in the plasma velocity. As the jet propagated deeper into the magnetosheath, the amplitude of the dynamic pressure and velocity enhancements decreased and the transitions between the ambient and jet plasma were smoothed out. Omelchenko et al. (2021) similarly studied jets associated with enhancements of density, velocity, and dynamic pressure in 3D simulation runs of the hybrid-PIC model HYPERS. They found that the amplitudes of the enhancements decrease with increasing distance downstream of the bow shock. Palmroth et al. (2021) conducted a statistical study of magnetosheath jets in four 2D Vlasiator simulation runs. Jets at the bow shock were found to be associated with enhanced density, velocity, dynamic pressure, and magnetic field strength, compared to the ambient magnetosheath plasma surrounding the jets, see Fig. 12. The enhancements decreased and converged toward the ambient magnetosheath fluctuation level with increasing distance downstream of the bow shock. Differences were observed in the enhancements depending on the solar wind parameters of the runs. With lower IMF strength, the density and magnetic field strength enhancements had higher amplitudes and the velocity magnitude decreased more slowly with distance from the bow shock. At the shock, the parallel and perpendicular temperatures inside the jets were lower than in the ambient magnetosheath, but the temperatures increased and converged toward that of the ambient magnetosheath further downstream of the bow shock. In a statistical study of Cluster observations, Echim et al. (2023) came to the same conclusion on perpendicular ion temperature increasing inside jets with decreasing distance to the Earth. Plaschke et al. (2020b) studied the alignment of magnetic field and jet velocity using observations from the four Magnetospheric Multiscale (MMS) spacecraft and found that the magnetic field inside jets tends to be more aligned with the jet velocity closer to the bow shock than farther away from it.

#### Evolution of Morphology

These simulation studies also investigated the evolution of the shape and size of magnetosheath jets. The single jet studied in Palmroth et al. (2018) was found to initially grow faster in the radial direction (with respect to the centre of the Earth), reaching a maximum radial size of 2.8 $R_{\mathrm{E}}$. At the time of maximum radial size, the transverse size was 0.8 $R_{\mathrm{E}}$. After the time of maximum radial size, the jet detacheed from the bow shock, its radial size abruptly decreasing to 1.2 $R_{\mathrm{E}}$ and its transverse size started increasing toward a maximum of 1.3 $R_{\mathrm{E}}$. The jet thus evolved into a rounder shape. With statistical analysis, Palmroth et al. (2021) studied the $x$-directional (along Sun-Earth line) extent and transverse size of jets as a function of distance downstream from the bow shock. They found that at the bow shock, jets were associated with a higher extent than transverse size, and that the average extent was larger in the runs with lower IMF strength. With increasing distance from the bow shock, the extent slowly decreased, while the transverse size increased, until the extent and transverse size became roughly equal. This agrees with the case study in Palmroth et al. (2018). Goncharov et al. (2020) used MMS observations to study the evolution of the characteristic sizes and shapes of jets. They found that jets were larger in the direction parallel to the plasma flow within the jet than in the direction perpendicular to it, and that both of these sizes increased with distance downstream from the bow shock. This result does not agree with the simulation studies of Palmroth et al. (2018, 2021).

#### Propagation

**Fig. 13:**
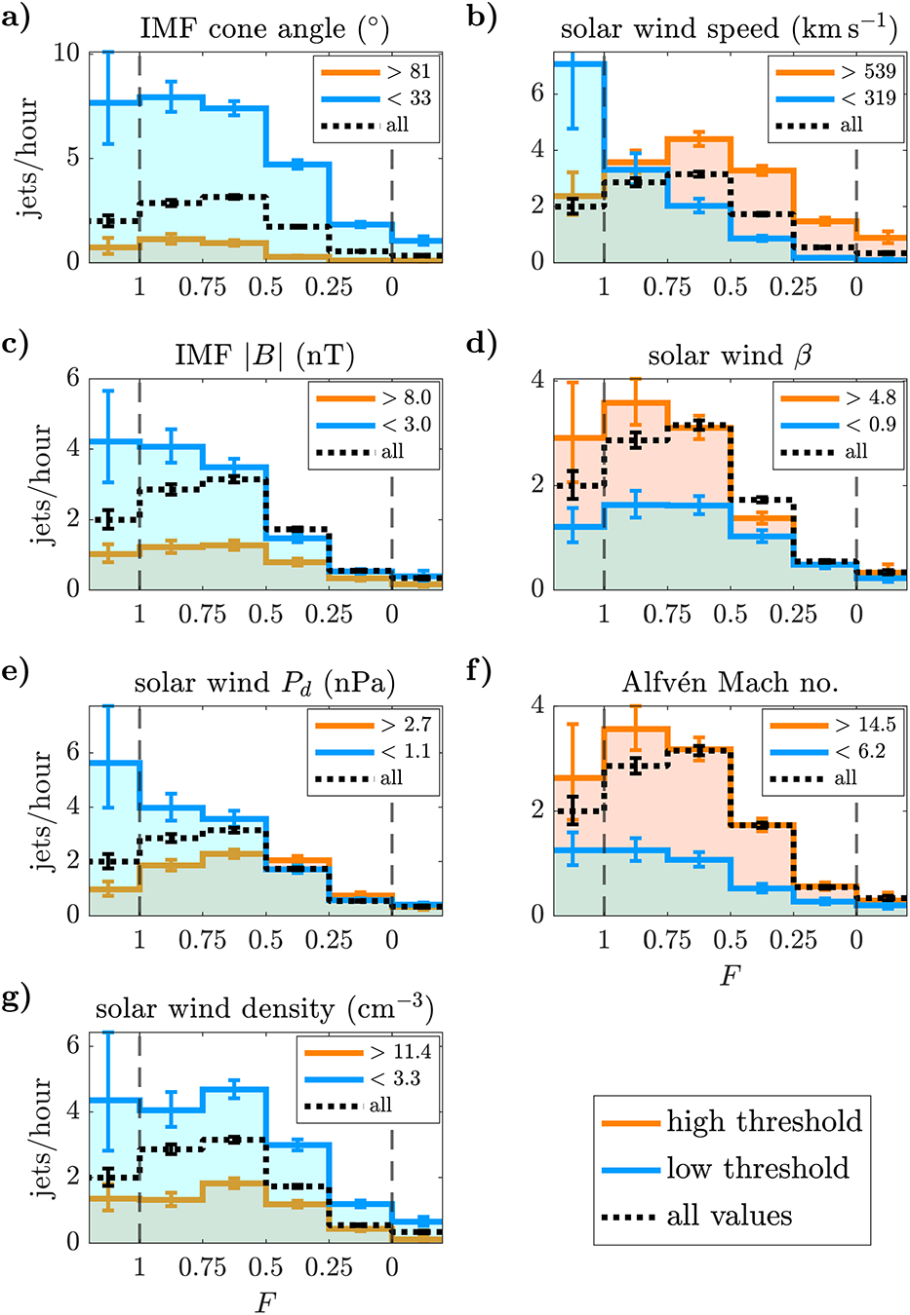
Histograms of jet observation frequencies by THEMIS at different distances from the bow shock. Locations in the magnetosheath are estimated from a model magnetosheath as a normalised distance $F$ where $F=1$ denotes the bow shock and $F=0$ the magnetopause. Observations are separated into two categories for each of the solar wind parameters (a) IMF cone angle, (b) solar wind speed, (c) IMF magnitude, (d) solar wind plasma $\beta $, (e) solar wind dynamic pressure, (f) solar wind Alfvén Mach number, and (g) solar wind density: values which exceed the 85th percentile of the parameter value (high threshold, orange), and values which are below the 15th percentile (low threshold, blue). For comparison, the histograms for all values are also shown (dotted). Image reproduced with permission from LaMoury et al. (2021), copyright by AGU

The most well-studied aspect of jet evolution is their propagation in the magnetosheath as a function of different solar wind and IMF parameters. LaMoury et al. (2021) used magnetosheath observations from the THEMIS spacecraft and solar wind observations from the OMNI data set to study jets under different solar wind conditions. They found that jets were more likely to be observed deeper in the magnetosheath when the IMF strength and cone angle were low, and when the solar wind speed and Alfvén Mach number were high and the solar wind density was low. The cone angle and solar wind speed were found to be the most important parameters controlling magnetosheath penetration depth, as seen in Fig. 13. Palmroth et al. (2021) found that the number of identified jets decreased with increasing distance downstream of the bow shock, and it decreased more rapidly when the IMF strength was higher (corresponding to lower solar wind Alfvén Mach number). They also found that as jets travelled deeper in the magnetosheath and their speeds decreased toward the magnetosheath flow speed, their propagation direction also tended to align with the magnetosheath flow when the IMF cone angle was high, but not when it was low. Similar to previous findings, Goncharov et al. (2020) also found that jets were observed more often near the bow shock, and that their propagation directions tended to align with the magnetosheath flow as they slowed down.

### Interaction with Ambient Plasma

As jets propagate through the magnetosheath, their interaction with the ambient plasma not only changes the properties of the jets, but also affects the surrounding magnetosheath plasma. Plaschke et al. (2017) investigated the interaction of 18 jet events with MMS measurements. They observed ambient plasma flowing perpendicular and opposite to the jet propagation (sometimes even flowing sunward in the GSE reference frame). They interpreted these results as a displacing motion of the plasma ahead and in the vicinity of jets.

**Fig. 14:**
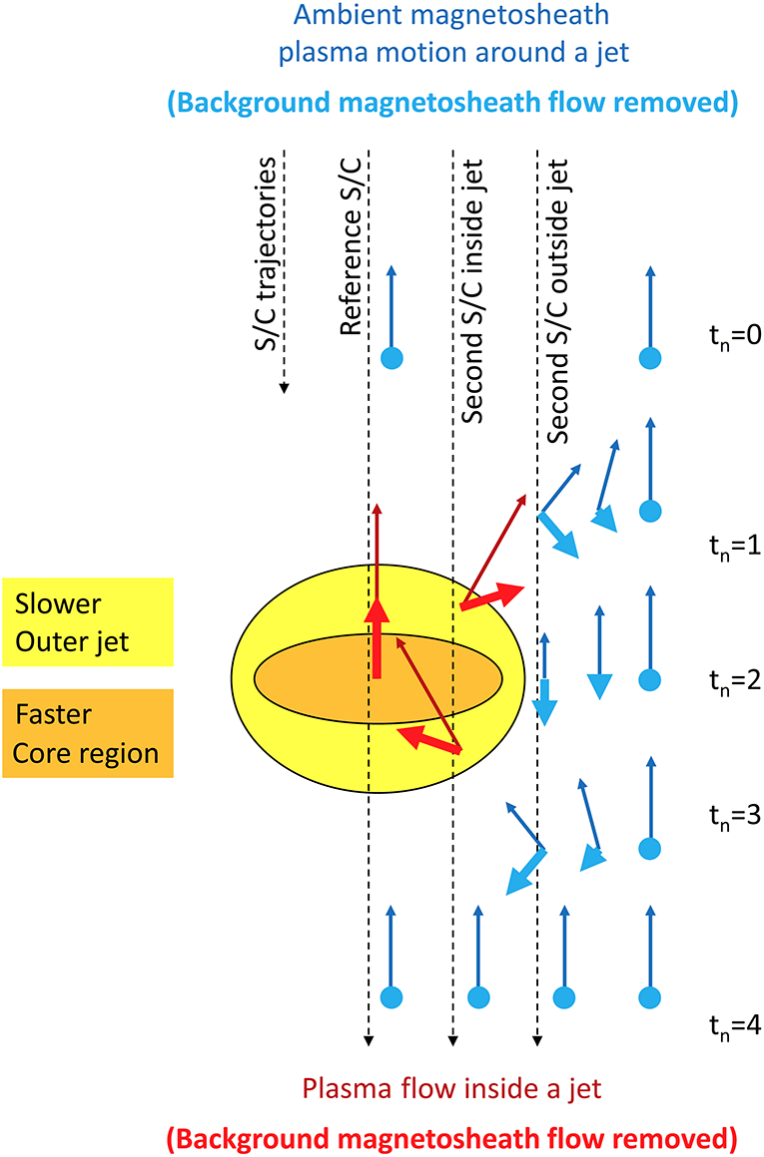
Diagram from Plaschke and Hietala (2018) depicting the flow of plasma in the vicinity of a magnetosheath jet. Red arrows show flow velocity within the jet, while blue arrows show the flow velocity outside the jet. Thin arrows show velocity in the laboratory frame, thick arrows in the plasma frame. The jet consists of a faster core region (orange) and a slower outer region (yellow). Image reproduced with permission from Plaschke and Hietala (2018), copyright by the author(s)

This idea was supported with a statistical study of several hundreds of jets by Plaschke and Hietala (2018). They used THEMIS spacecraft observations and studied the plasma motion inside and outside jets. For this purpose, the authors utilised measurements from pairs of spacecraft oriented along a line nearly perpendicular to the jet propagation direction. They found diverging plasma flows ahead of and converging flows behind the cores of the jets. Additionally, they observed that the ambient plasma in the vicinity of the jets was moving opposite to the propagation direction in the rest frame of the magnetosheath, but observed no sunward median flows. Together with previous results from simulations (Karimabadi et al. 2014), Plaschke and Hietala (2018) concluded that the jets plough through the magnetosheath and stir the ambient plasma, as illustrated in Fig. 14. The fast jets accelerate the slower plasma ahead of them and push it out of the path of propagation. The wake left behind is then filled by the magnetosheath plasma. The authors speculated that the properties of jets should also play a role. The scale size perpendicular to the propagation should be especially important, because this should correlate with the amount of plasma pushed aside.

**Fig. 15:**
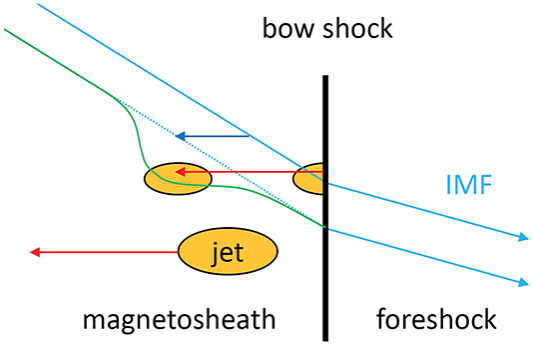
Illustration of how the plasma motion of a high-speed jet (red arrow) through slower ambient plasma (blue arrow) modifies the magnetic field in the magnetosheath (green line). Image reproduced with permission from Plaschke et al. (2017), copyright by AGU

Plaschke et al. (2017) additionally observed that the magnetic field inside of jets aligned with the propagation direction. Figure 15 shows how the frozen-in magnetic field is dragged with the magnetosheath plasma by the fast jet. As jets move towards the magnetopause, they straighten the magnetic field lines, so that they become more aligned with the jet propagation direction.

Plaschke et al. (2020b) investigated this behaviour further with a statistical study of several thousand jets with MMS spacecraft. The authors conducted a superposed epoch analysis of the angle $\phi $ between the velocity and the magnetic field and found that jets do modify the magnetic field and align it to an extent with the velocity. This alignment is greater for faster jets and for jets closer to the bow shock. Nevertheless, the overall alignment is small and has no strong dependency on IMF cone angle, IMF strength, solar wind velocity, density, dynamic pressure, or Mach numbers. Individual jets can look very different, because the variability of $\phi $ inside of jets is slightly larger than outside and of the same order of magnitude as the alignment.

Jets can also excite the Kelvin-Helmholtz instability (KHI) if the velocity jump between jet and ambient plasma is large, as Guo et al. (2022) showed in their global hybrid simulation. The KHI can grow and cause a meandering of the jets in their simulation runs.

Just as the velocity jump can cause the KHI, the other interactions mentioned above can also give rise to instabilities at the jet boundaries. These instabilities can grow over time and evolve into waves within and in the vicinity of the jets. The next subsection will discuss the latest results on this topic.

#### Waves Inside of Jets

Previous studies (Gunell et al. 2014; Eriksson et al. 2016) have reported observations of whistler mode and lower hybrid frequency waves, as well as structures like current sheets, within jets. Karlsson et al. (2018) used MMS spacecraft observations in the magnetosheath and THEMIS-B observations in the upstream solar wind to investigate six jets in detail and found support for the previously reported lower hybrid and whistler waves. The authors observed emissions below the electron cyclotron frequency in the power spectral density of the magnetic and electric field, interpreting them as electron whistler waves. While the waves were visible around and within the jet, the activity was higher inside jets. The authors estimated the upper limit for the energy radiated by these waves and found that the time required to radiate away the kinetic energy of a jet would be approximately 22 hours. Therefore, the authors concluded that these waves are likely unimportant for the evolution of jets as their travel time in the magnetosheath is far shorter, on the order of minutes. They also observed lower hybrid frequency waves within jets, with higher activity at the edges. This is consistent with earlier reports by Gunell et al. (2014) who suggested these waves were generated at density gradients at the edges of jets. Additionally, Karlsson et al. (2018) observed electrostatic oscillations above the electron gyrofrequency, which they speculated to be broadband electrostatic noise. In contrast to earlier studies, the authors observed low-frequency, compressional electromagnetic waves with higher amplitudes within the jets. These waves were only visible for jets in the quasi-parallel magnetosheath, suggesting a relation to foreshock ‘10 s waves’ (Blanco-Cano and Schwartz 1997), or ‘30 s waves’, which are known to be transmitted through the bow shock (Turc et al. 2023). Karlsson et al. (2018) estimated the upper limit for the radiation process, finding that the low frequency waves could radiate away the kinetic energy of a jet in roughly 7 minutes and could play an important role in its evolution. However, how much these low frequency waves may affect jets depends on their origin.

Recently, Krämer et al. (2023) investigated the wave activity at three jets in more detail. They used the high resolution burst mode measurements of the MMS spacecraft. The authors not only reported different types of waves, but also explained possible formation mechanisms for them. They observed whistler waves in agreement with previous studies and found a butterfly-shaped pitch angle distribution in almost all occurrences. They suggested these distributions as the source of the waves, though were not able to explain where they came from.

**Fig. 16:**
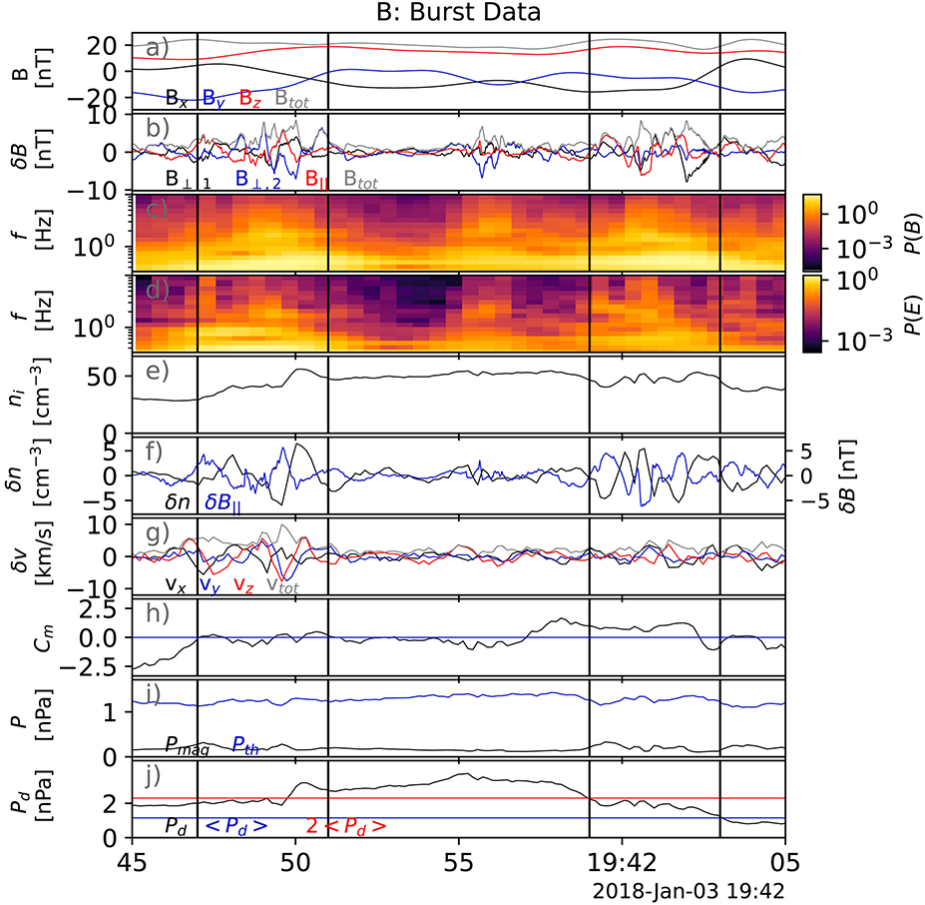
Example measurements of 1 Hz waves in the burst mode data of the MMS spacecraft. From top to bottom background magnetic field (a), AC magnetic field (b), magnetic field spectrum (c), electric field spectrum (d), ion density (e), variations in ion density and in parallel magnetic field (f), variations in ion velocity (g), instability threshold for mirror mode (h), magnetic and thermal pressure (i) and (background) dynamic pressure (j) are shown. The vertical black lines indicate the 1 Hz wave packets at the edges of the jet. Image reproduced with permission from Krämer et al. (2023), copyright by the author(s)

Prior to these observations, Gunell et al. (2014) had speculated that electron beams at jet boundaries act as a generation mechanism, but could not investigate this further. While Krämer et al. (2023) did not observe increased electric fields needed for the electron beam, they could not rule out this explanation due to the limitation of the plasma instrument. The authors also observed broadband electrostatic wave activity (0.2–2 kHz). Contrary to the suggestion of Karlsson et al. (2018), most of these waves were not solitary waves but localised wave packets. They suggested that these oscillations were electron acoustic mode waves. The small fraction of electrostatic solitary waves had a duration of just a few milliseconds. Moreover, they had a small spatial extent or were heavily damped, since they were observed from only 1 or 2 spacecraft. Although they were unable to determine the source of the solitary waves, Krämer et al. (2023) speculated that one wave might be an ion phase space hole (phase-space structures where trapped ion density is lower at the center than at the rim, e.g., Aravindakshan et al. 2021). They also observed 1 Hz waves associated with density gradients and magnetic field rotations at the edges of the jets as shown in Fig. 16. Comparison with basic wave modes showed that they exhibit properties of multiple modes and that simple approximations as used in textbooks could not describe these waves. As reported in previous studies, the authors observed wave activity between 5 and 200 Hz, which most likely stemmed from lower hybrid waves. Since not all observed waves can be described as lower hybrid waves, they suggested that the approximations are not appropriate for all of them. Krämer et al. (2023) also observed 0.2 Hz waves similar to the 0.1 Hz waves reported by Karlsson et al. (2018). Complementary to these works which studied mainly the quasi-parallel magnetosheath, Blanco-Cano et al. (2020) studied the quasi-perpendicular magnetosheath, and observed with MMS not only mirror modes but also transverse oscillations with periods of 10–20 s within neighbouring jets.

Katsavrias et al. (2021), on the other hand, observed compressional pulsations immediately after the passage of a jet with the THEMIS-A spacecraft. The pulsations were visible in two frequency bands in the Pi2 range (7.6–9.2 mHz and 12–17 mHz). The authors suggested that the pulsations were fast Alfvén waves. They concluded that these waves were locally generated since they were only visible shortly after the jet. The observed frequencies upstream were actually below the Pi2 frequency range, but waves upstream of the bow shock are expected to have different frequencies compared to waves downstream. Pi2 pulsations are triggered by changes in the magnetic field, therefore, Katsavrias et al. (2021) concluded that the fast jet abruptly changed the magnetic field, as suggested by Plaschke et al. (2017), leading to the formation of Pi2 pulsations. As discussed in Karlsson et al. (2018), these waves are energetically weak compared to the plasma kinetic energy, but they could facilitate the exchange of energy between jets and the ambient plasma surrounding them. This could be the mechanism through which jets slow down as they approach the magnetopause. The authors also observed similar oscillations in the magnetosphere, although they did not provide an explanation for how the pulsations propagate through the magnetopause.

### Shocks and Particle Acceleration

The simulations of Karimabadi et al. (2014), a previous study already discussed in Plaschke et al. (2018), suggested that when supermagnetosonic jets compress the magnetosheath plasma, a bow wave can build ahead of the jet. Liu et al. (2019) observed such bow waves using THEMIS data. Of the 2859 jet events in the list from Plaschke et al. (2013), 364 (∼13%) events were found to be associated with a bow wave. The criterion used to identify bow waves required that the jet was supermagnetosonic in the spacecraft frame and that enhancements of magnetic field strength and density as well as a change in the plasma flow direction were observed at the leading edge of the jet. In some cases the bow waves may have steepened into shocks (Liu et al. 2020), however, no distinction was made between these cases. As they were presumably in different stages of development, all events were called shock-like events.

Liu et al. (2020) examined probability distributions of plasma and magnetic field solar wind parameters and compared the distributions for shock-like jet events and non-shock events. The authors showed that shock-like events were more likely to occur during periods of large solar wind dynamic pressure (but not high density or solar wind velocity alone), low magnetic pressure and therefore high plasma $\beta $ (thermal pressure had no influence), high Alfvén Mach number, and a small IMF cone angle. Also, the probability of observing shock-like events close to the bow shock was smaller than for non-shock events. Therefore, it is likely that some time and space is needed for the bow wave to form. Liu et al. (2020) also examined the probability distributions of the maximum ion and electron energies. Independent of the ambient magnetosheath temperature, shock-like events exhibited higher electron and ion energies. Additionally, the electron energy flux was on averaged enhanced by a factor of 2 above ∼100 eV compared to the background and non-shock events. This confirmed the results from Liu et al. (2019, 2020), who conducted case studies of shock-like events. Liu et al. (2019) examined one event and showed that ions and electrons are accelerated by bow waves. Using a single-particle model, the ion acceleration was explained by ion reflection at the bow wave. Liu et al. (2020) examined three more events to characterise the electron acceleration more precisely. They showed that some electrons were energised by shock drift acceleration and subsequently reflected, while others continued moving downstream of the bow wave, resulting in a bidirectional motion. The additional energisation of suprathermal electrons and ions in jet driven bow waves implies that jets can increase the efficiency of electron and ion acceleration at planetary bow shocks. Pandey et al. (2024) used data from the Mars Atmosphere and Volatile EvolutioN (MAVEN) mission to study jet-associated waves in the Martian magnetosheath. They found mirror modes, solitary waves, and electron acoustic waves with properties similar to those of jets in Earth’s magnetosheath. However, they also observed a double layer inside a jet, which has not been observed in jets at Earth. Double layers could contribute to changing the plasma properties inside Martian jets as they are efficient at accelerating particles.

How jet-associated bow waves contribute to electron acceleration was studied with 1D Monte Carlo test-particle simulations, where the bow wave was modelled as a MHD shock (Vuorinen et al. 2022). Their results suggested that a collapsing magnetic trap forming between the bow wave and the magnetopause can explain the energy flux increases of ∼100 eV electrons to around 10 keV (in extreme cases to a few hundred keV) with shock drift acceleration. The best fit between model and observations was found with weak scattering. A significant parameter for the efficiency of the acceleration was the speed of the bow wave, but magnetosheath conditions in general also contributed. Additionally, the geometry between bow wave and magnetic field affected the acceleration, because it affected the interaction time. For bow waves closer to the magnetopause, the acceleration was stronger.

The formation and evolution of bow waves in front of jets was studied with a 2D hybrid-PIC model by Ren et al. (2024b). The authors found that a bow wave can form if there is a magnetic structure in the path of a supermagnetosonic jet with field lines nearly perpendicular to the bulk velocity of the jet. Investigating one such jet-associated bow wave in particular, they found that the jet compressed the field in front of it, causing the bow wave to have enhanced magnetic field and density. In turn, the jet was slowed down, and after some time it could no longer drive the bow wave. The bow wave dissipated after 15–25 ion gyroperiods.

Eriksson et al. (2016) also reported parallel electron acceleration during a jet event. They observed a strong current sheet inside of a jet, concluding that the sheet probably formed locally due to a velocity shear related to the jet, but no clear signatures of reconnection could be found during this event. They proposed that the accelerated electron beam most likely formed due to an electrostatic potential difference along the ambient magnetic field.

### Discussion

Recent studies of magnetosheath jets have shed light on their macro- and microphysical properties as well as their evolution. Spacecraft studies using VDF data have investigated the temperature anisotropy inside jets as well as other properties of the VDFs. These studies found that there are several kinds of jets with different properties, and some jets are associated with multiple ion populations. In addition, studies of the plasma moments also found evidence for different kinds of jets, and that jets can modify the plasma flow and magnetic field surrounding them. Additionally, other spacecraft studies found that jets can be associated with many different kinds of waves, and that they can drive bow waves in the magnetosheath, potentially causing particle acceleration.

Magnetospheric simulation studies, on the other hand, have mainly focused on the evolution of jets in terms of properties, morphology, and propagation. They found that the plasma in jets is thermalised and jets align their propagation with the ambient flow. Jets also change shape as they travel deeper into the magnetosheath. Some spacecraft studies compared jets observed at various distances downstream of the bow shock, coming to conclusions that jets grow in size with distance from the bow shock. This disagrees with simulations that showed that extent of the jet decreases while only the transverse sizes increases.

The summarised studies show that the process of understanding the properties and behaviour of magnetosheath jets has resulted in the discovery that jets are even more complex than previously thought. The internal structure and microphysical properties of jets, such as changing temperature anisotropy and non-bi-Maxwellian VDFs, require investigation with kinetic models. The interaction between jets and ambient magnetosheath plasma, e.g. generation of bow waves that accelerate electrons and modification of plasma flow and magnetic field, can have far-reaching effects on other parts of the magnetosheath or even the magnetosphere. The non-Maxwellianity of VDFs as well as temperature anisotropy are also potentially favourable for the generation of waves through instabilities. Jets in the quasi-parallel and quasi-perpendicular magnetosheath appear to have differing properties, but the differences are still unclear and not all studies agree on what these differences are.

Spacecraft observations have limitations when studying the evolution of magnetosheath jets, because a single spacecraft cannot directly observe the evolution of an individual jet over large distances or time scales, even though statistics derived from large databases can be a powerful inference tool. Furthermore, spacecraft studies that rely on investigating plasma moments rather than full VDFs give an incomplete picture of the physics of jets, as the velocity distributions associated with jets can be highly non-Maxwellian and agyrotropic. Hybrid-kinetic models are useful for directly simulating the evolution of jets as well as their properties. But those hybrid simulations are currently limited in other ways, such as restrictions to two spatial dimensions, being noisy, or simulating a downscaled magnetosphere.

In the future, spacecraft constellations consisting of a larger number of satellites than current missions could be used to directly observe jet evolution across different scales. More advanced instrumentation will give better resolved VDFs at higher cadence, aiding in the study of jet properties. On the simulation front, unscaled, 3D global, low-noise, hybrid-kinetic simulations will give a more realistic picture of magnetosheath jets as a whole.

## Magnetospheric Impacts and Global Dynamics

This section discusses the latest findings on the effects of magnetosheath jets on the magnetospheric and ionospheric system. The enhanced dynamic pressure of magnetosheath jets causes local indentations of the magnetopause. Magnetosheath jets and jet-driven waves can trigger fast and Alfvén modes at the magnetopause which can transmit information deeper in the magnetosphere and to the ionosphere. Plaschke et al. (2018) discussed a large range of studies reporting magnetosheath jet impacts on the magnetopause. Jets can for example trigger magnetopause motion (e.g., Hietala et al. 2009; Amata et al. 2011), impulsive penetration of magnetosheath plasma in the magnetopause (Gunell et al. 2012; Karlsson et al. 2015) and localised flow enhancements in the ionosphere (Hietala et al. 2012). In addition, they can drive other instabilities which can lead to subsequent wave modes. Plaschke et al. (2018) suggested that magnetosheath jets could transmit information from the foreshock to the magnetosphere which may be significant for solar wind-magnetosphere interactions during solar quiet times. However, they also noted that jets are not the dominant transfer mechanism as they do not show a correlation with southward-oriented IMF conditions. More recent studies have investigated the role of magnetosheath jets in triggering magnetic reconnection at the magnetopause. In addition, ground-based instrumentation has been utilised to study magnetosheath jets impacts on the ionosphere and ground-based magnetic responses. These new studies are presented and discussed in the following.

### Impact Rates at the Magnetopause

Magnetosheath jets can influence the magnetosphere by impinging onto its outer edge, the magnetopause. The first step towards understanding the importance of jets on magnetospheric dynamics is therefore to estimate how often jets impact the magnetopause. Plaschke et al. (2016) provided the first estimations of such impact rates. Their methodology and results have been considered in detail in the previous review by Plaschke et al. (2018). Since then, Plaschke et al. (2020a) continued this work and obtained a log-normal distribution for the transverse sizes of jets close to the magnetopause (as reviewed in Sect. 3.1.3) and used the log-normal distribution to produce an updated estimate for the impact rates of jets at the magnetopause.

**Fig. 17:**
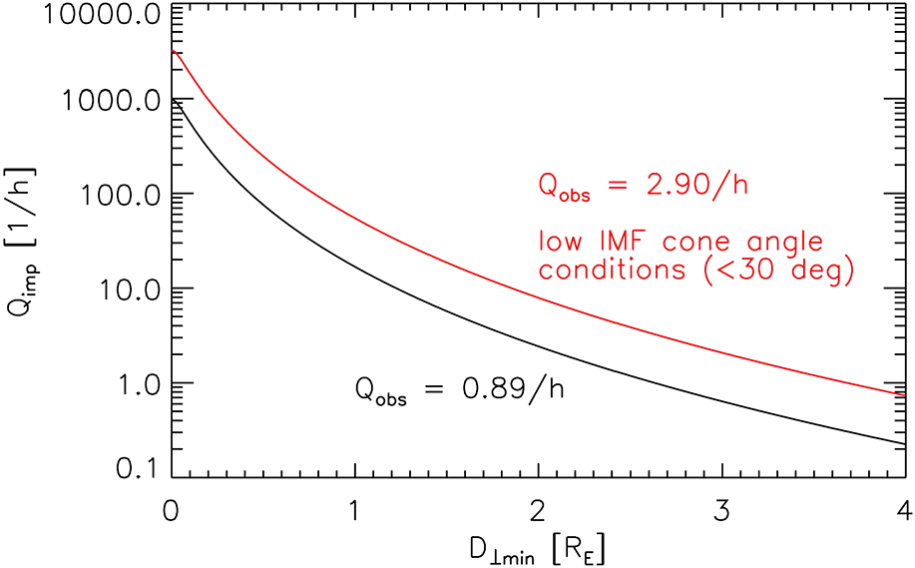
Estimated magnetopause impact rates $Q_{\mathrm{imp}}$ of jets as a function of the minimum perpendicular diameter $D_{\perp \mathrm{min}}$. The black (red) line shows impact rates corresponding to the observed jet occurrence rate of $Q_{\mathrm{obs}} = 0.89/$h ($Q_{ \mathrm{obs}} = 2.90/$h) during all (low, $<30^{\circ}$, IMF cone angle) conditions. Image reproduced with permission from Plaschke et al. (2020a), copyright by AGU

The estimates of Plaschke et al. (2020a) for the numbers of jets impacting a circular reference area $A_{\mathrm{ref}}=102\,R^{2}_{\mathrm{E}}$ on the subsolar magnetopause are shown in Fig. 17. They found impact rates of jets with $D_{\perp }> 2\,R_{\mathrm{E}}$ to be $Q_{\mathrm{imp}}=2.4/$h overall and $Q_{\mathrm{imp}}=7.9/$h during low ($<30^{\circ}$) IMF cone angle conditions. These rates are about 17% smaller than those reported by Plaschke et al. (2016), showing that the previous results obtained for large-scale jets hold reasonably well, and that previous conclusions stating that these large geoeffective jets are very frequent still hold. The new model works much more reliably in the regime of smaller jets, and they estimate that thousands of jets, of which most are small, may hit the magnetopause per hour. Vuorinen et al. (2019) also calculated jet impact rates during different IMF cone angle conditions using the Plaschke et al. (2016) model. Their reported rates remain largely unchanged when using the new model: e.g., jets with $D_{\perp }> 1$ $R_{\mathrm{E}}$ are estimated to impact the magnetopause reference area at a rate of 0.9/min during low ($<30^{\circ}$) IMF cone angles, 0.4/min during oblique ($[30^{\circ},60^{\circ}]$) IMF cone angles, and 5.1/h during high ($>60^{\circ}$) IMF cone angles. Suni et al. (2021) reported an impact rate of about 26/h using the global hybrid-Vlasov code Vlasiator under radial IMF conditions.

As discussed in Sect. 3.2.3, LaMoury et al. (2021) reported that other solar wind parameters, in addition to the IMF cone angle, influence the jet observation rates close to the magnetopause, and this is due to a combination of formation and propagation effects. In particular, jets are much more likely to reach the magnetopause when the solar wind speed is high: jets are almost five times more common near the magnetopause when solar wind speed is greater than 600 km/s compared to when it is less than 400 km/s. Similarly, jets are almost three times more common when the solar wind density is lower than $5\,\mathrm{cm}^{-3}$ compared to when it is larger than $20\,\mathrm{cm}^{-3}$. In the future, the magnetopause impact rates of jets should be estimated for different types of solar wind upstream conditions to better understand when jets are most likely to be geoeffective. Furthermore, future estimates should account for the mass and energy carried by the jets.

### Magnetic Reconnection at the Magnetopause

**Fig. 18:**
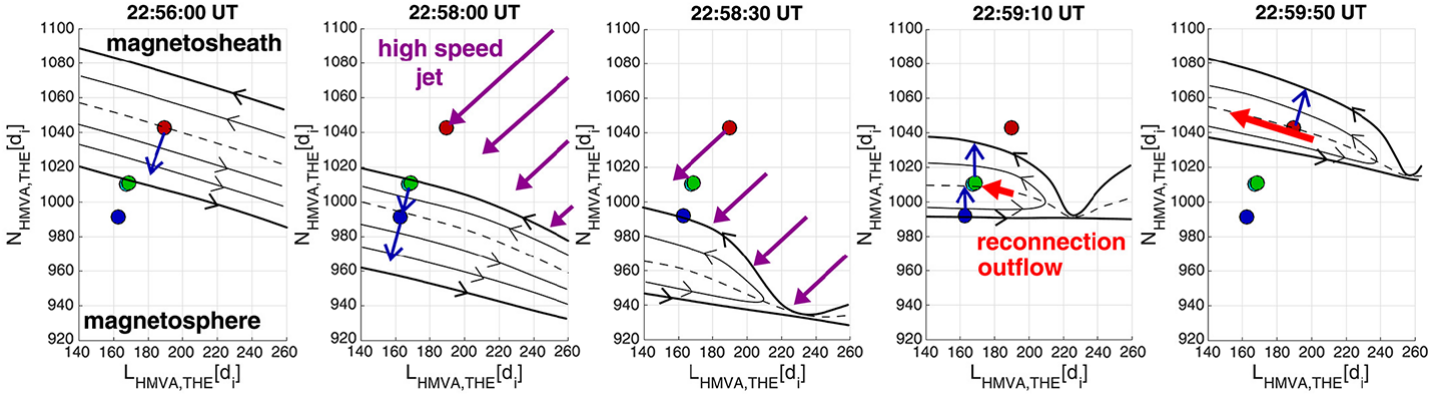
Schematic depicting how the high dynamic pressure of a jet was thought to compress the magnetopause layer, thus triggering reconnection at the magnetopause where there previously was none. Circles denote THEMIS spacecraft positions. Image reproduced with permission from Hietala et al. (2018), copyright by AGU

While suggestions of a potential causal link between jets and reconnection at the magnetopause have existed for some time (e.g., Plaschke et al. 2016), studies specifically examining this have only recently come to fruition. The onset of magnetic reconnection is understood to be influenced by the $\beta $-asymmetry, magnetic shear angle, and system scale size (Swisdak et al. 2010). Jets may be able to trigger or suppress magnetopause reconnection by changing the local plasma environment at the magnetopause with respect to these conditions. This was demonstrated in a study by Hietala et al. (2018), which utilised the THEMIS probes in a string-of-pearls formation when they passed through the dayside magnetopause. Spacecraft entered the magnetosheath from the magnetosphere, and did not see reconnection occurring at the magnetopause, despite favourable conditions in terms of $\beta $ and magnetic shear. They concluded that reconnection was prevented by the magnetopause layer being unusually thick. While in the magnetosheath, a jet reached the magnetopause. Shortly after, the magnetopause advected back over the spacecraft showing signs of a reconnection exhaust. It was determined that the large dynamic pressure impulse exerted on the magnetopause by the jet was sufficient to compress the magnetopause current sheet so that it was able to reconnect. This process is illustrated in Fig. 18. This study represents the only direct observation to date of a jet triggering reconnection at the magnetopause.

Other studies, however, have inferred jets triggering reconnection by connecting jet observations and ground effects. Nykyri et al. (2019) used a multi-spacecraft conjunction to deduce that jet-driven reconnection at the magnetopause contributed significant flux loading to the magnetotail and eventually led to the onset of a substorm. Most notably, this occurred during a period of weakly northward, quasi-radial IMF, i.e., a condition in which reconnection at the dayside magnetopause would not traditionally be expected to a large extent. Despite this, jets were observed in the magnetosheath containing pulses of strongly southward (negative) $B_{\mathrm{Z}}$ which, they inferred, provided the necessary antiparallel fields required for reconnection onset.

**Fig. 19:**
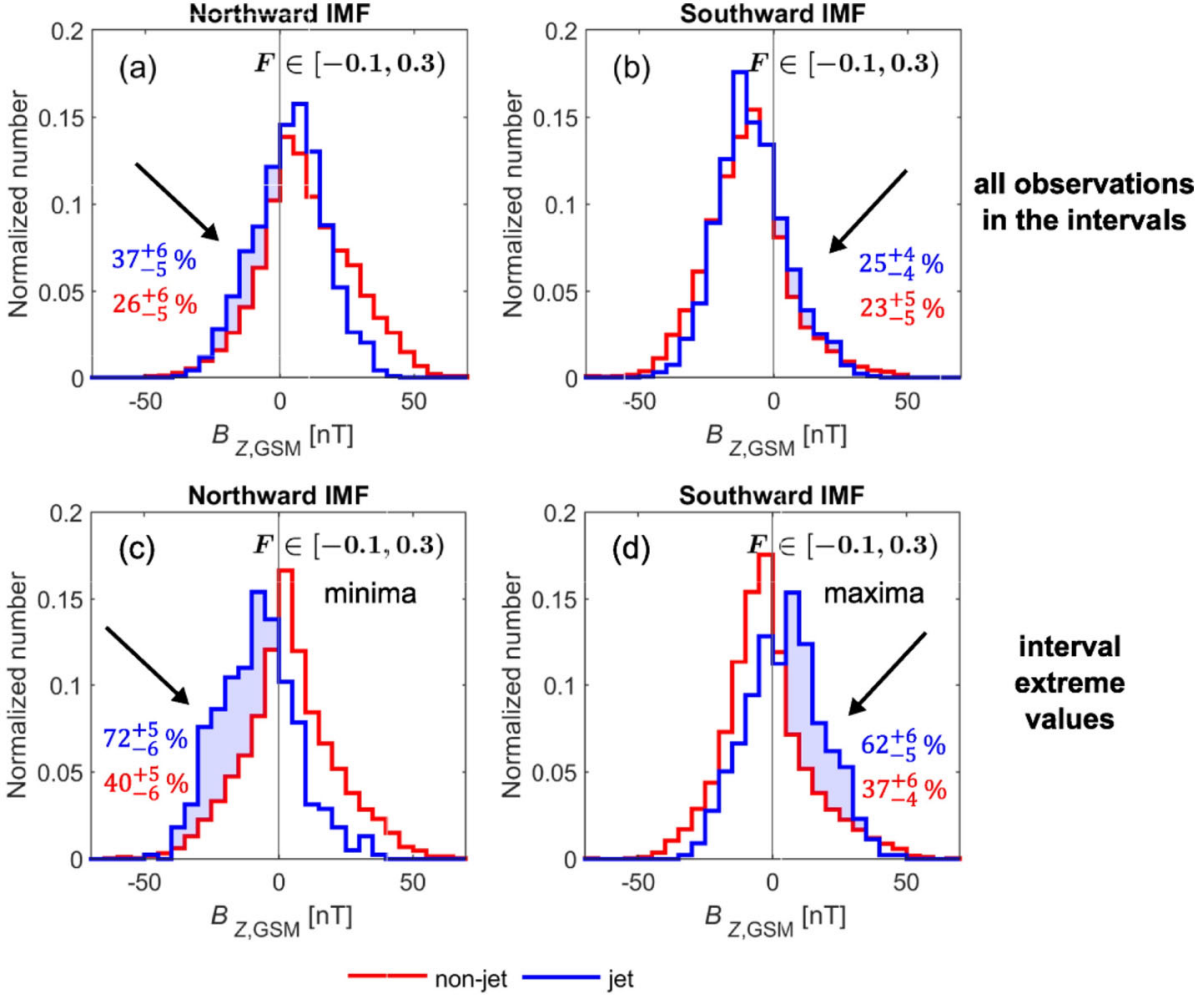
Histograms showing the Z-GSM component of magnetic field in jets (blue) versus non-jet magnetosheath intervals (red). Panels a) and b) use all plasma from within the intervals, while panels c) and d) use only the extreme points of each interval (the point most oppositely directed to the IMF $B_{\mathrm{Z}}$). Left column: northward IMF. Right column: Southward IMF. Image reproduced with permission from Vuorinen et al. (2021), copyright by the author(s)

The idea that jets may be able to trigger reconnection during northward IMF was further investigated by Vuorinen et al. (2021). They conducted a statistical study (using the same THEMIS data set as Plaschke et al. 2013) of jets near the magnetopause and found that the strong southward pulses such as those seen by Nykyri et al. (2019) were present in around 13% of jets. They also showed that jets were significantly more likely to contain pulses of $B_{\mathrm{Z}}$ oppositely directed to the upstream IMF than non-jet magnetosheath intervals under similar solar wind conditions. This is examined in Fig. 19, a key result of that study. When considering all magnetic field data within jet intervals, they found that $25^{+4}_{-4}$% of plasma observed during periods of southward IMF had a GSM $B_{\mathrm{Z}}$ pointed northward, while $37^{+6}_{-5}$% of jet plasma during times of northward IMF contained southward $B_{\mathrm{Z}}$. These are marginally higher proportions of oppositely-directed $B_{\mathrm{Z}}$ than seen in similar non-jet intervals (Figs. 19a and b). They went further, however, by examining the extreme portions of each interval. They saw that jets were significantly more likely to contain an opposite polarity pulse than the non-jet magnetosheath, finding that $62^{+6}_{-5}$% of jets observed during southward IMF contained some amount of northward $B_{\mathrm{Z}}$, while $72^{+5}_{-6}$% of jets observed during northward IMF contained some amount of southward-pointing $B_{\mathrm{Z}}$ (Figs. 19c and d). From their statistics, they were able to conclude that jets not only increase the potential for reconnection triggering during northward IMF, but also make it possible for jets to suppress ongoing reconnection during times of southward IMF. This means that the majority of jets contain some amount of magnetic field which could potentially alter reconnection at the magnetopause from what would typically be expected from the IMF orientation, and that this likelihood is greater than in the non-jet magnetosheath during similar solar wind conditions.

Jet driven local reconnection has been also suggested to be connected to the formation of diamagnetic cavities. Burkholder et al. (2021) found that most diamagnetic cavities were observed in the location consistent with formation due to the low latitude or high-latitude reconnection for the prevailing IMF, but some events were also found which were consistent with neither. The authors suggested that such events could be potentially related to jets since they can cause transient reconnection sites.

**Fig. 20:**
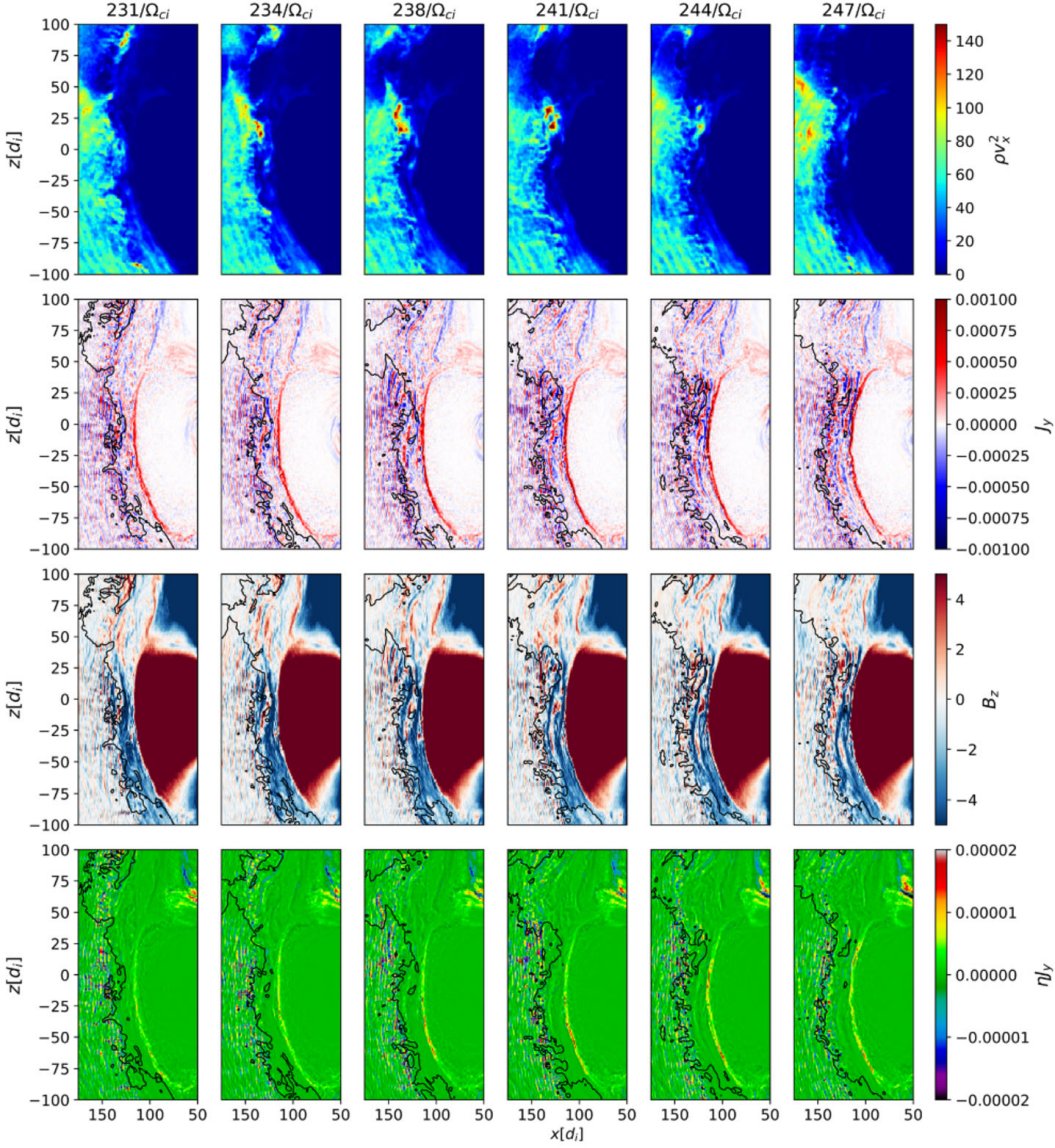
Slice of 3D hybrid simulation of dayside magnetosphere, shown at several timesteps (columns). Quantities shown are (top to bottom): X-directed dynamic pressure, current density, $B_{\mathrm{Z}}$, and the y-component of the resistive term in Ohm’s law. Image reproduced with permission from Ng et al. (2021), copyright by the author(s)

The role of jets in magnetopause reconnection has also been explored in recent simulation studies. Ng et al. (2021) analysed 3D hybrid simulations of the dayside magnetosheath during quasi-radial southward IMF, using a simulation run also studied by Omelchenko et al. (2021). Figure 20 shows a slice of the 3D simulation at several time steps. They inferred that jets were involved in triggering localised and bursty reconnection, contributing to the formation of FTEs. They concluded that jets are able to modulate magnetopause dynamics on top of the quasi-steady X-lines created by prolonged southward IMF. This is in agreement with previous simulation studies, such as the 2D global hybrid-kinetic simulations performed by Karimabadi et al. (2014). In this study, jets were seen to significantly disturb the magnetopause environment, leading to the onset of reconnection and the formation of FTEs.

### Magnetopause Motion

Local changes in the dynamic pressure in the magnetosheath will result in a deformation of the magnetopause. Magnetosheath jets are defined as dynamic pressure enhancements, therefore the direct response of a jet impacting the magnetopause is a local inward motion of the latter. This inward motion of the magnetopause has been previously observed (Amata et al. 2011; Hietala et al. 2012; Archer et al. 2012). How extreme these local distortions can be, was discussed by Němeček et al. (2023). Furthermore, in recent years, studies have also shown that jets not only lead to local disturbances of the magnetopause but they act as a seed for disturbances in the magnetosphere and on the ground.

Archer et al. (2019), using an interval included in the study of Dmitriev and Suvorova (2015), confirmed with observations the magnetopause surface eigenmode motion – a surface wave of the magnetopause standing between the northern and southern ionospheres. This surface eigenmode was triggered by an isolated jet impinging the magnetopause. The eigenmode has long been hypothesised (Chen and Hasegawa 1974) and has even been simulated (Hartinger et al. 2015). The surface eigenmode has an expected fundamental frequency of or below 2 mHz (Archer and Plaschke 2015; Hartinger et al. 2015). Archer et al. (2019) observed both the fundamental frequency and the first harmonic of the magnetopause surface eigenmode after a jet caused the initial perturbation of the magnetopause. A follow-up study showed that the magnetopause surface eigenmode can persist as a stationary wave on the dayside magnetopause (9-15 h magnetic local time) by propagating against the magnetosheath flow (Archer et al. 2021). The mode can also act as a seed for surface waves that are advected further tailward, which grow in amplitude through the Kelvin-Helmholtz instability and subsequently couple to other ULF wave modes that propagate into the magnetosphere. In general, ULF waves are known to transport energy across the magnetosphere and have been associated with auroral activity. However, so far it remains uncertain how often these magnetopause surface waves occur and what role jets have as drivers of these surface waves.

Processes other than surface waves can cause the magnetopause to move. A study by Escoubet et al. (2020) showed that multiple magnetosheath jets can cause magnetopause indentations independent of each other using a conjunction between Cluster and MMS. The authors compared the observed magnetopause normal with the expected normal from models and found significant differences. In their study, the area that was affected by magnetopause motion due to jets was larger than 10 R_E_. In addition, Němeček et al. (2023) found that predicted magnetopause radial distances showed large deviations from the observed magnetopause positions for a near radial IMF and low dynamic pressure in the solar wind. The reported magnetopause displacement was 4 R_E_ in the sunward direction, while the earthward displacements was around 1–2 R_E_. The authors suggested that these localised displacements of the magnetopause are caused by magnetosheath jets, either by a single intense jet or by a series of less intense jets impacting the magnetopause. Similar results were found in a study on the magnetopause response to the impact of a single jet (Ma et al. 2024). The magnetopause was found to react with an “Indentation-Rebounce-Relaxation” sequence with spatial scales of 0.5–3.2 R_E_.

**Fig. 21:**
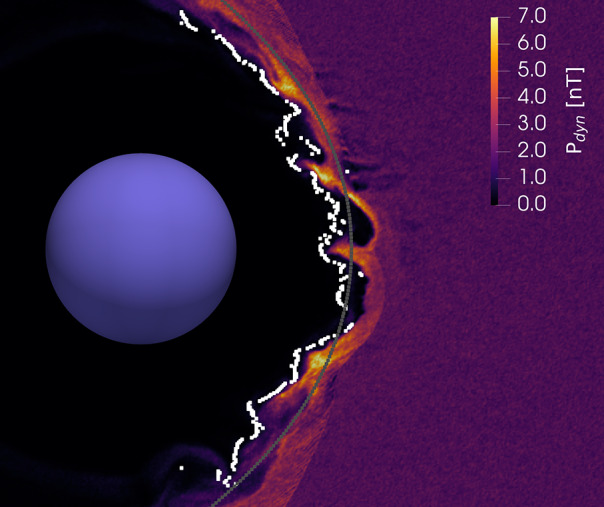
Simulation results using the Amitis simulation (Fatemi et al. 2017), a 3D, global, hybrid simulation, showing the deformation of the magnetopause (white dots) due to the impact of magnetosheath jets in the $xy$-plane during a run with quasi-parallel, southward IMF ($15^{ \circ}$ IMF cone angle and 5 nT IMF strength, simulation run R1S in Fatemi et al. (2024)). The magnetopause was determined using the maximum magnetopause current density. For comparison, the statistical magnetopause position proposed by Shue et al. (1997) is shown in grey. Magnetosheath jets can be identified by the increase in dynamic pressure and subsequently disturb the magnetopause. The inner boundary (blue sphere) is at 4.7 $R_{ \mathrm{E}}$

Archer et al. (2019, 2021) and Escoubet et al. (2020) showed that jets impacting the magnetopause can create a globally disturbed magnetosphere. When the dayside magnetosphere is largely downstream of a quasi-parallel shock, multiple jets can impact the magnetopause at different locations (Escoubet et al. 2020) and cause extreme deviations from magnetopause models (Němeček et al. 2023). Furthermore, a single jet can cause magnetopause surface waves. In both cases the magnetopause cannot be considered a smooth surface. This implies that magnetopause models downstream of the quasi-parallel bow shock represent only the average magnetopause location, as the boundary is generally very disturbed. Figure 21 displays such a disturbed magnetopause using simulation results from the Amitis model (Fatemi et al. 2017) during a quasi-parallel IMF. Amitis is a 3D, global, hybrid model of the Earth’s magnetosphere. The position of the magnetopause (white dots), which was determined by the peak current density, varies significantly from the statistical magnetopause position proposed by Shue et al. (1997). Jets triggering ULF waves are well known (Hietala et al. 2012; Archer et al. 2013b,a; Norenius et al. 2021; Wang et al. 2022), but these studies have assumed that these effects are localised. Findings of Archer et al. (2019, 2021) and Escoubet et al. (2020) however suggest that jet disturbances may be more global.

### Inner Magnetospheric, Ionospheric and Ground Effects

Jet impacts on the magnetopause can excite auroras in the dayside ionosphere. In particular, a connection between a discrete auroral form called ‘throat aurora’ and jets has been hypothesised, based on the observation that these auroras are seen more frequently during low IMF cone angle conditions (Han et al. 2017). Throat auroras extend equatorward (aligned close to the north-south direction) from the equatorward edge of the east-west auroral oval, and they are observed in the midday sector, where this edge is believed to map to the open-closed magnetic field line boundary at the magnetopause. Han et al. (2016) showed that throat auroras are associated with magnetosheath-like particles precipitating into the ionosphere. Han et al. (2018) used concurrent observations of MMS and ground-based auroral images to show that throat auroras could be temporally and spatially (via MMS footpoint) linked to localised magnetopause displacements. Fast earthward flows were also observed in the magnetosheath, but their connection to the indentations was not clear. The observations also indicated that the width of the auroral form is proportional to the duration of the transient excursion to the magnetosheath.

**Fig. 22:**
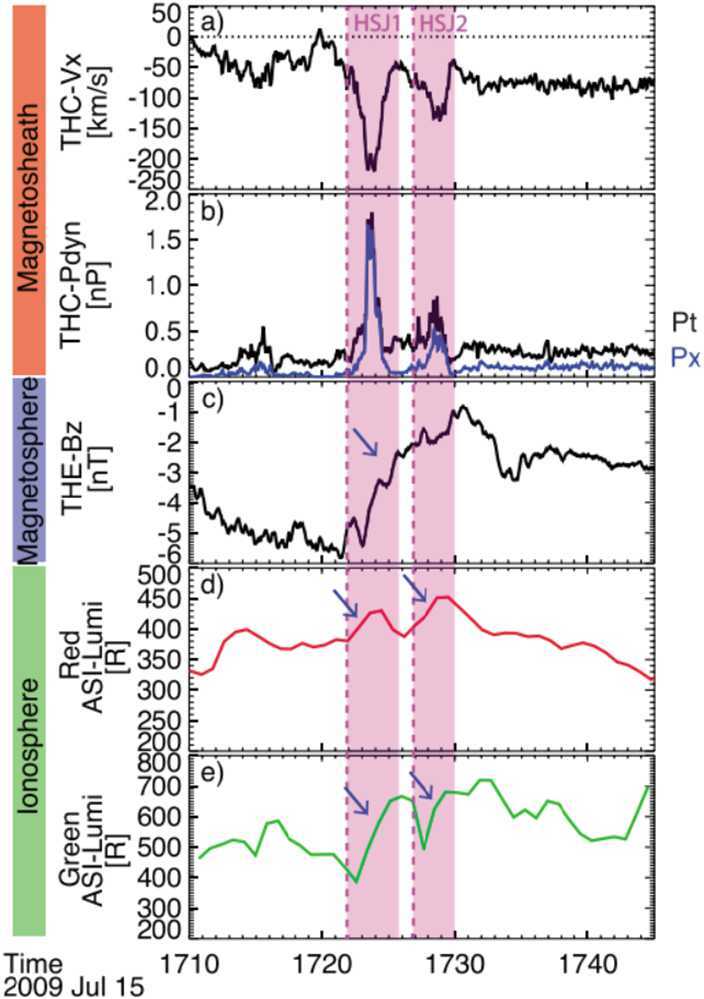
THEMIS C observations in the magnetosheath: (a) GSM $X$ velocity component and (b) total (black) and earthward (blue) dynamic pressure. THEMIS E measurements in the magnetosphere: (c) GSM $Z$ magnetic field component. South Pole auroral luminosities averaged over $10^{\circ}$–$30^{\circ}$ magnetic longitude: (d) red line and (e) green line. The magenta dashed lines and shaded regions denote the beginning and duration of the jets, respectively. The blue arrows highlight the magnetospheric and auroral responses coinciding with these jets. Image reproduced with permission from Wang et al. (2018), copyright by AGU

Wang et al. (2018) utilised dayside conjuctions between THEMIS spacecraft and an all-sky imager (ASI) at the South Pole Station, Antarctica, during 2008–2010 to investigate the auroral response to jets during eight suitable events. Both discrete and diffuse auroral brightenings were observed in all of the events. Figure 22 shows an overview of one of the events on July 15, 2009. THEMIS C observed jets in the magnetosheath, THEMIS E measured a change in magnetospheric magnetic field due to compression, and both the red (discrete aurora) and green (diffuse aurora) auroral emissions were enhanced. The average size of the diffuse auroral forms corresponded to around 800 km when mapped into the ionosphere and to a 3.7 $R_{ \mathrm{E}}$ azimuthal width in the equatorial plane. The east-west propagation direction of the auroral brightenings was consistent with the magnetosheath background flow direction, suggesting that the magnetosheath flow pushed the magnetopause deformation towards its propagation direction. In most cases, local magnetospheric ULF waves were found, indicating that jet-related magnetospheric compression was indeed localised. The authors did not find throat auroras in these events, as the discrete auroras were brightenings in the auroral oval. They proposed that these discrete brightenings were due to enhanced electron precipitation in the form of field-aligned currents, which could be excited when jets compress the magnetopause and flow shears or travelling convection vortices arise. According to the authors, the diffuse auroral brightenings were most likely related to localised magnetopause compression caused by jets, which can lead to increased electron anisotropy and energy, growth of whistler waves, and enhanced precipitation. The authors concluded that auroral signatures of jets are similar to shock aurora, except smaller in spatial and temporal extent.

Nishimura et al. (2020) presented a 25-min interval of MMS observations in the magnetosheath with earthward jets and concurrent auroral images observed at the Kjell Henriksen Observatory ASI in Longyearbyen, Svalbard. Their findings are in line with those of Wang et al. (2018). The jets were associated with diffuse auroral brightenings. In addition, a discrete brightening on the existing auroral oval was observed. A throat aurora was also visible in this interval, but as there was no diffuse auroral brightening associated with it, Nishimura et al. (2020) argued that this throat aurora was not caused by magnetosheath jets. The connection between auroral brightening and jets was further investigated by Qiu et al. (2024). The authors suggested that ULF waves accelerate electrons through parallel electric fields causing the typical “inverted-V” structure. These accelerated electrons subsequently cause auroral brightening.

Ng et al. (2022) used 3D global hybrid simulations to study the magnetospheric cusp regions under quasi-radial IMF conditions. They found that when the cusp is close to the quasi-parallel shock, foreshock turbulence and transient structures in the magnetosheath can perturb the region. In their simulations, jets caused density enhancements near the cusp by compressing plasma ahead of themselves. Importantly, in a run with southward IMF, on-going quasi-steady magnetopause reconnection allowed for these density enhancements to enter the cusp. Such enhancements in the cusp may lead to enhanced particle precipitation.

Dmitriev and Suvorova (2023) investigated the magnetospheric and upper-atmospheric effects of jets that were observed by THEMIS on 12 July 2009. They linked the formation of these jets to IMF rotational discontinuities. The authors found evidence of magnetospheric compression and impulsive penetration of suprathermal magnetosheath-like plasma into the magnetosphere as jets interacted with the magnetopause. Dst index reached 7 nT around the time of a strong earthward jet and this variation lasted for ∼16 min. They argued that this suggested a travelling jet interacting with multiple points on the magnetopause. Evidence of associated high-latitude ionospheric precipitation was found in POES satellite measurements together with signatures of enhanced ionospheric currents on the dayside sector, corresponding to magnetic disturbances of up to 100 nT, seen in SuperMAG ground-magnetometer data and a 30% increase in ionisation at high latitudes found in GPS data.

**Fig. 23:**
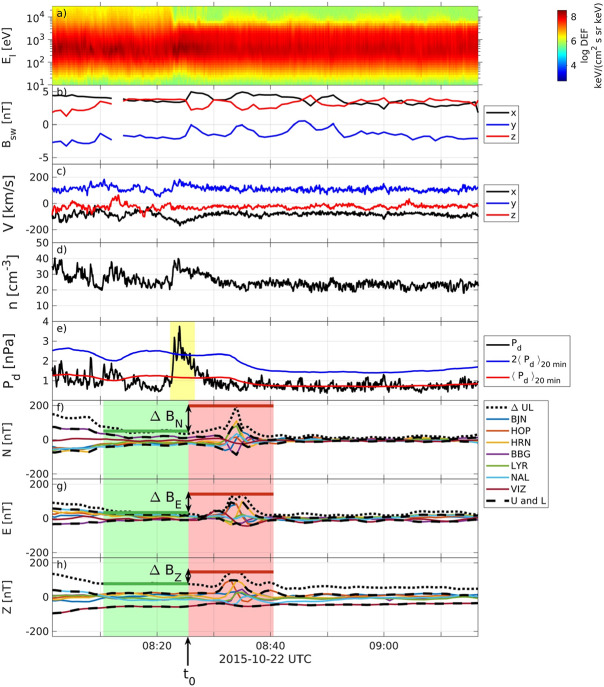
A magnetosheath jet observed by MMS1 on Oct 22, 2015, and its ULF ground response observed by seven SuperMAG ground-magnetometer stations within 2,000 km of the estimated footpoint. (a) MMS1 ion energy spectrogram, (b) OMNI IMF components (GSE), (c) MMS1 ion velocity (GSE), and (d) MMS1 electron density. Panel (e) includes MMS1 dynamic pressure observations (black) and 100% (red) and 200% (blue) of its 20 min running average, with the magnetosheath jet highlighted in yellow. (f–h) $N$ (north), $E$ (east), and $Z$ (up) components of the SuperMAG ground magnetic field observations. Image reproduced with permission from Norenius et al. (2021), copyright by the author(s)

Norenius et al. (2021) used MMS observations of jets and investigated their ground-based ULF wave response from observations of the SuperMAG network on the northern hemisphere. They propagated jets to infer the first closed field line the jet would impact and its ionospheric footpoint and investigated ground responses at stations within 2,000 km of this footpoint. They presented a statistical study of 65 events along with a case study of an example event on 22 October 2015. Figure 23 presents the observations of this example event: MMS1 observations from the magnetosheath (panels a & c–e), OMNI IMF observations (panel b), and SuperMAG ground magnetometer observations (panels f–h). The observed jet (highlighted in yellow in panel e) was believed to have been formed due to an IMF rotational discontinuity. There was a 120 s delay from MMS1 measuring the centre of the jet to the first ground magnetic response, and the responses peaked at an amplitude of 130 nT around 10 min after the jet. The responses were different at the seven eligible stations, and some of them could be well-fitted with damped oscillations with an average frequency of around 2 mHz. In the statistical study, the median time delay was 190 s, the median $e$-folding time of the response was 370 s, and the median frequency was 1.7–1.9 mHz. The response was typically stronger for jets of longer duration and of higher dynamic pressure, but these explained only around 20% and 10% of the variance, respectively. Most jets had relatively weak magnetic responses of $\sim 20$ nT. The authors estimated that the largest observed disturbance of 158 nT would cause variations of $dB/dt \sim 40$ nT/min on average. They argued that while meaningful power grid impacts have occurred in conditions less than $\sim 100$ nT/min, jets are most likely not harmful for power grids.

Wang et al. (2022) continued the investigation of Pc5 (1.66–6.66 mHz, or periods 150–600 s; extended up to 700 s in their analysis) waves, i.e., ground magnetic disturbances caused by magnetosheath jets, with a statistical study of 664 events using THEMIS 2008–2011 and 2016 data in conjunction with SuperMAG and THEMIS GMAG data. These events belonged to either of two categories: recurrent jets, where at least three jets were observed with <12 min separation between consecutive jets, and isolated jets, where only one jet was observed. They presented two example events, one of each category, which indicated that the frequency of the ground magnetic response following a recurrent jet event may be determined by the recurrence time of jets. In their statistical analysis, the authors found that 37% of all observed jet events could trigger ground magnetic ULF waves that were distinct from those supposedly arising from a random distribution of responses for non-geoeffective jets. When they only considered jets with earthward dynamic pressure larger than 2 nPa, the proportion of geoeffective jets was 46%. These percentages correspond to responses in the northward component, for which the proportion of geoeffective jets was found to be the largest. Additionally, jets were found to be more likely geoeffective the higher their peak earthward speed and dynamic pressure. Finally, 50% of jets observed close to the magnetopause were geoeffective in comparison to 25% (8 out of 32) of jets observed close to the bow shock.

While the effect of jets on radiation belt dynamics have not been studied in detail, Liou et al. (2023) suggested that jets could cause microinjections into the magnetosphere which could act as a seed for relativistic electrons in the radiation belt. The authors showed how 62% percent of the energetic electron microinjections were created by drift mirror instability (DMI) within dayside magnetospheric boundary layer. However, the non locally DMI driven microinjection events could be related to foreshock phenomena, such as high-speed jets.

### Discussion

Magnetosheath jets are known to frequently impact the magnetopause where they cause large but localised indentations. For jets with radii >2 R_E_ the impact rate has been estimated to be 8 jets/hour during low cone angle conditions and 3 jets/hour overall (Plaschke et al. 2020a). These impact rates indicate that the dayside ionosphere is frequently disturbed by magnetosheath jets. Small jets are expected to interact with the magnetopause at even higher rates. These magnetopause disturbances have been associated with a range of consequences in the inner magnetosphere and ionosphere such as ground ULF waves or auroral activity, and can act as the last trigger for a substorm onset.

Recent work has bolstered the connection between jets and reconnection at the magnetopause, but the exact form of the link remains unclear. The statistical study of $B_{\mathrm{Z}}$ near the magnetopause by Vuorinen et al. (2021) demonstrated that jets are likely to be able to alter reconnection at the magnetopause based on their inherent fluctuating magnetic field orientations. It is well-established, however, that there are other controlling factors in the onset of reconnection. Theoretical and simulation work by Swisdak et al. (2010) showed that reconnection may be suppressed by a diamagnetic drift of the X-line when a current sheet is subject to a pressure gradient and guide field (out of plane component, i.e., not perfectly antiparallel), as is common at the magnetopause. If the lateral drift speed exceeds the reconnection outflow speed, reconnection is suppressed. This condition manifests as a relation concerning not only the magnetic shear angle at the current sheet, but also the difference in plasma $\beta $ across it. Observations in the solar wind and at the magnetopause have provided strong evidence as to the validity of this theory (e.g., Phan et al. 2010, 2013). Therefore, in order to fully assess the potential of jets to trigger or suppress reconnection at the magnetopause, more complete studies of the controlling parameter space must be performed.

Despite promising evidence from predictive statistics and simulations, direct observations of jets altering reconnection at the magnetopause are still extremely limited, with the case study by Hietala et al. (2018) being the only example to date. With more case studies, we may gain a greater understanding of the role of jets in the wider magnetospheric system. The extent to which jet-driven reconnection contributes to global dynamics is not yet known, though recent efforts have shed light on the localised effects. It may be the case that the more common effect of jets on reconnection is, like the indentations produced on the magnetopause, local rather than global. This view is supported by simulation work in which jets are seen to be involved in bursty reconnection and the production of FTEs. Tentative corroborating observational evidence for this has been provided by Kullen et al. (2019), who saw examples of isolated FTEs forming during low IMF cone angle conditions, suggesting that these may be linked to the impact of jets.

That is not to say that jet-driven reconnection cannot contribute to global dynamics. In the study by Nykyri et al. (2019), jets were linked to substorm onset, though they were not thought to be solely responsible. The study event was preceded by a period of southward IMF, during which flux was loaded into the tail. The system stopped short of substorm onset, however, upon the rotation of the IMF to a weakly northward configuration. The interpretation is, therefore, that the pulses of southward $B_{\mathrm{Z}}$ brought by jets provided the final stimulus that allowed the substorm to occur, not that the entire sequence was jet-driven. It is possible, therefore, that jets may modulate the magnetospheric system without driving it, adding uncertainty and stochasticity to idealised models. Further investigation into the role of jets in global dynamics remains to be conducted.

Recent observations have shown that jets can excite both discrete and diffuse auroral forms. The increased electron precipitation into the ionosphere might be linked to jet-related flow shears and localised compression of the magnetopause, which enhance FACs and the growth of magnetospheric whistler waves, respectively. Simulations also suggest that jet impacts near the cusp regions together with ongoing magnetopause reconnection can cause strong density enhancements in the cusp, potentially leading to enhanced particle precipitation. For the first time, the ground magnetic disturbances following jets have been measured and studied statistically. A significant proportion of $\sim 40\%$ of jets have been found to trigger ground ULF waves. These direct ionospheric and ground-based measurements provide convincing evidence that jets are geoeffective, and the significance of these effects on the solar wind-magnetosphere interaction should be determined. While the impact of a single jet is localised and not likely to be significant when compared to larger transient structures, their overall contribution must arise from their frequency.

The possible connection between throat auroras and magnetosheath jets remains somewhat unclear. Although throat auroras have been linked to magnetopause indentations, it has not been explicitly established how these indentations are produced or that they would be related to jets. Recently, Han (2019) suggested a conceptual model of throat aurora, in which they form due to magnetopause reconnection controlled by factors both inside and outside of the magnetosphere. They suggested that jets do not cause them directly, but can influence their formation indirectly by exciting so-called stripy diffuse auroras. The model suggests that existing diffuse aurora precipitation leads to an ionospheric polarisation electric field, which is mapped to the magnetopause and causes an earthward drift of a reconnection site, leading to signatures of throat aurora. Wang et al. (2018) and Nishimura et al. (2020) showed evidence of diffuse auroral brightenings associated with jets. While not the only contributing factor to diffuse aurora, jets could thus increase the occurrence of throat aurora during low IMF cone angles, as has been observed by Han et al. (2017). Recent observational evidence by Han et al. (2019) suggests that throat auroras are related to magnetopause reconnection, giving further support for the model of Han (2019). Whether jets can drive throat auroras on their own by deforming the magnetopause and triggering reconnection remains an open question. Overall, auroral images are an important tool for understanding how jets influence the magnetosphere and the ionosphere. In the future, they could be used for inferring properties of the incident jets.

While significant progress has been made in recent years, there still exist several barriers to assessing the impact of jets on the magnetospheric and ionospheric systems. The geoeffectiveness of jets has been mostly assessed by works studying only a few events at a time with the exception of ULF waves on the ground. Studies with only few events focus on strong jet events that are often associated with IMF discontinuities. While these event studies provide important insight, they neglect the cumulative effect of smaller jets. Limited instrumentation coverage on the ground, or limited conjunction between spacecraft in the magnetosheath and magnetosphere introduces additional difficulties for statistical studies on ground effects. Furthermore, tracking jets from the magnetosheath into the ionosphere can be challenging and relies on magnetospheric models. This introduces additional difficulties in distinguishing magnetospheric disturbances caused by jets from those arising from other sources. Plaschke et al. (2018) noted that the magnetospheric and ionospheric disturbances have been previously associated with the foreshock or the solar wind. The authors also discussed that jets cannot be the dominant mode for the solar wind-magnetosphere coupling, but whether their contribution is significant during quiet times should be determined.

In the future, larger statistical studies might be able to reveal the frequency of individual magnetospheric signatures. In addition, spacecraft conjunctions could be used to trace the magnetospheric disturbances from the magnetopause to the ground. Furthermore, the effects of bursty reconnection at the magnetopause on the magnetosphere during non-southward IMF is an undergoing subject of investigation. Future studies could also investigate the amount of energy and momentum transferred into the magnetosphere during a jet impact.

## Open Questions

In this section we briefly reflect on the open questions posed by Plaschke et al. (2018) making an assessment of the extent to which they have been answered. We dwell on those left outstanding, and pose new questions and future challenges that have emerged in light of recent discoveries.

### Open Questions on Formation and Occurrence

Significant progress has been made in understanding jet formation in the past years, though many new questions have arisen from the gained knowledge.

In the previous review, Plaschke et al. (2018) posed questions concerning the variety of mechanisms that may produce jets, and whether observed jet properties may be related to their origin. Different generation mechanisms have been proposed using observations (Kajdič et al. 2021; Raptis et al. 2022b) and simulations (Preisser et al. 2020; Omelchenko et al. 2021; Suni et al. 2021, 2023) in recent years. The jet properties connected to those mechanisms still need to be investigated statistically to conclude whether the origin source can be distinguished unambiguously.

The role of solar wind parameters and solar wind transient phenomena on jet formation and occurrence has been addressed in multiple works (Vuorinen et al. 2019; LaMoury et al. 2021; Koller et al. 2022, 2023; Vuorinen et al. 2023a,b; Koller et al. 2024). However, no systematic work on how solar wind turbulence affects jets has been performed.

Below we pose remaining, overarching questions that break up into smaller sub-questions in each topic.

**What is the role of variable solar wind and turbulence in jet formation?**

This question is an extension of the open issues posed in the previous review. The solar wind comprises different structures from large to small scales and is very variable in nature. What role does this variability play in jet formation? For example, how steady does the IMF need to be for jet formation? Future simulations including variable solar wind input might help understanding how jets get formed.

Discontinuities, which are known to form jets, also often arise in the solar wind. Whether the formation of these jets is the result of foreshock spatial and temporal variations or due to the formation of mesoscale upstream foreshock structures (e.g., foreshock bubbles or HFAs) has not yet been investigated in detail. Large-scale solar wind discontinuities can produce boundary jets, whether those are connected to foreshock compressional boundaries is so far unclear in observations. What would be the scale size of these discontinuities needed to form jets? Can large-scale solar wind structures and discontinuities within them cause differences in the magnetosheath, resulting in boundary jets as defined by Raptis et al. (2020a) and can we analyse them statistically?

Turbulence in the solar wind is a cross-scale phenomenon which is injected at larger scales, cascading down to macro and micro scales. The role of solar wind turbulence in the formation of jets is as yet unexplored. Can turbulent solar wind be statistically connected to jet occurrence? Our understanding of the interplay of turbulence and shocks dynamics has progressed somewhat (e.g., Rakhmanova et al. 2020; Trotta et al. 2022; Gedalin 2023), therefore it may be reasonable to assume that jet formation and occurrence could be similarly affected by turbulence. Upcoming simulations might also help in answering these questions.

**How does jet formation compare at different shocks?**

Different environments can be used as ‘laboratories’ to understand the universality of jet formation. With the detection of jet in the Martian (Gunell et al. 2023) and Jovian magnetosheaths (Zhou et al. 2024), as well as behind interplanetary shocks (Hietala et al. 2024), we can now begin to evaluate jets in different environments. So far, these investigations have chiefly concerned their scale sizes. We can also explore the formation of jets under different shock strengths and plasma conditions. The upcoming BepiColombo mission may allow us to assess the potential for jets to form in the magnetosheath of Mercury. Whether jets form at cometary shock environments is still open for investigation. Are there time and spatial scale limitations, restricting potential jet formation to specific scales? If so, what would be the implication for previous large statistical results?

**What is the role of jet formation at weak shocks and magnetosheath flanks?**

The connection of jets with magnetosheath flanks is so far under-investigated. What are the properties of jets formed at the flanks are they borne of the same formation mechanisms? What would be the implication for jets existing in environments with high cone angles (Jupiter, Saturn)? Do flank jets follow the overall flow or maintain their velocity and direction? What would be the implication for jet formation at similar low mach number shocks, e.g., behind interplanetary shocks?

**Can we find a different approach to jet definitions, connected to their formation?**

Recent studies of both simulations and observations revealed that different jet criteria heavily influence which jets are detected in which spatial region of the magnetosheath. Until now, all criteria have been based on thresholds in either velocity, density, or pressure of ions. Each criterion is useful for different science questions, however, the overlap between detected jets in different criteria is often small. Can we define jets based on other properties? Can we set definitions that disentangle jets coming from different formation processes? One possible way would be to analyse VDFs, as jets often show a mix of two populations of plasma. Thus, a search for non-Maxwellian distributions might help in defining a jet. Using ion temperature anisotropy or plasma stability may also help to define specific types of jets. The influence of heavy ions on jet formation has also not yet been investigated.

The formation of the majority of jets appears to be closely tied to the physics of collisionless shocks. Several jet formation mechanisms explain different aspects of the quasi-parallel shock, however, all these interpretations might point to the same effect with different interpretations (shock reformation, impacting SLAMS, subsequent ripples). Non-stationarity of shocks and the transmission of waves and transients through the shock are the important factors that we need to understand to develop a complete theory of jet formation at shocks.

### Open Questions on Properties and Evolution

In their previous review, Plaschke et al. (2018) posed several questions concerning the properties and evolution of magnetosheath jets, and we now have answers to some of these questions. Plaschke and Hietala (2018) investigated the flow patterns of plasma around jets using THEMIS data, while Plaschke et al. (2020b) studied the alignment of jets with the velocity and magnetic fields using MMS data. LaMoury et al. (2021) conducted a statistical study using THEMIS and OMNI data to investigate what solar wind conditions are conducive for jets to propagate through the entire magnetosheath to the magnetopause. Palmroth et al. (2018, 2021) used 2D hybrid-Vlasov simulations to study how jet properties change with increasing distance from the bow shock, while Omelchenko et al. (2021) did the same with 3D hybrid-PIC simulations.

Though progress has been made in understanding jet properties and evolution, many questions remain unanswered to some degree, while some have changed. Here, we explore some of these questions.

**Are there any features shared by all magnetosheath jets?**

The studies presented in this review have found that not all magnetosheath jets have the same properties. Some display isotropic Maxwellian VDFs, some are non-Maxwellian and anisotropic. Should the latter of these be considered to consist of multiple particle populations? Some jets show correlation between velocity and density and magnetic fields, while some do not. Jets found in the quasi-parallel magnetosheath are sometimes different from those found in the quasi-perpendicular magnetosheath, sometimes they are similar. This raises a question of whether there are some properties aside from dynamic pressure enhancements that are similar across all jets identified with the same criteria, hinting at a common microphysical generation process, or whether they are entirely different phenomena that all display dynamic pressure enhancements. Future studies of jets in environments other than the Earth’s magnetosheath could shed light on these questions.

**How do jets interact with and exchange energy with their surroundings?**

Recent studies have also discovered that jets slow down and change shape as they travel deeper into the magnetosheath. Due to the magnetosheath plasma being collisionless, these changes must be driven by non-collisional processes that allow for the exchange of momentum and energy between jets and their surroundings. The studies presented here have found waves both inside and around jets, as well as dragging and pushing of the ambient magnetosheath plasma and magnetic field by jets. Making quantitative estimates of the energy and momentum exchange through these processes requires additional work, however. The exchange rates require knowledge about the 3D sizes and shapes of jets, which are still quite unclear, although good progress has been made on this topic in recent years. Also unclear are the effects of electrons, due to most studies relying on ion measurements only. More sophisticated studies of how jets change over time require more multi-spacecraft missions.

**What effects do jets have on the magnetosheath?**

Closely connected with the previous question is the question of how jets affect the magnetosheath. Does the non-Maxwellianity and anisotropy of some jet VDFs lead to the generation of waves through instabilities, and how do these waves propagate and modify the magnetosheath? Can jets drive turbulence in the magnetosheath and, if so, how significant is this phenomenon? How exactly do jets generate bow waves, and how significant are these bow waves in accelerating particles? Do jets contribute to the thermalisation of the quasi-parallel magnetosheath and, if so, how? As with the interaction between jet and surroundings, studies of jet effects on the magnetosheath will also benefit from future multi-spacecraft missions.

### Open Questions on Magnetospheric Impacts

Plaschke et al. (2018) posed the questions on the role magnetosheath jets play in global dynamics of the magnetosphere. Since then, evidence has been found that magnetosheath jets could alter reconnection at the magnetopause (Hietala et al. 2018) and jets have been associated with a substorm onset (Nykyri et al. 2019). Furthermore, a significant proportion of jets has been found to contribute to ground ULF wave activity in the dayside magnetosphere (Norenius et al. 2021; Wang et al. 2022). The ionosphere is affected by jets through auroral brightening (Wang et al. 2018) and disturbances in the ionospheric current systems are monitored through AE and Dst indices (Dmitriev and Suvorova 2023). While some of these effects have been studied statistically, the occurrence rates and the dependence on jets properties has not. ULF waves can be directly triggered through the local disturbance of the magnetopause, however other effects might need a chain of physical processes to be triggered. We have also learned that trains of jets enhance the ground ULF activity, but we know little about other jet properties that enhance or suppress magnetospheric effects. Therefore, we propose the following overarching questions to be investigated in the future.

**What is the significance of magnetosheath jets to magnetospheric and ionospheric dynamics?**

How much energy and momentum do jets transfer into the magnetospheric-ionospheric system? Where is this energy deposited? What is the role of magnetosheath jets in (bursty) reconnection? And how does (bursty) reconnection affect the magnetosphere? Do jets pre-condition the magnetosphere and magnetopause? What is the significance of jets to solar wind-magnetosphere interaction, particularly during quiet times? Do jet impacts influence the radiation belts?

The effect of jets on the radiation belts has not yet been studied, but it is another mechanism that could increase the energy content in the magnetosphere. Also, little work has been done on how extended periods of radial IMF modifies the geospace system. Multiple jets impacting the magnetopause cause larger amplitudes in ground ULF waves (Wang et al. 2022). Jet occurrence is known to increase with steady IMF conditions (Archer and Horbury 2013), how do trains of jets modify the properties of the geospace system?

**What are the physical processes involved?**

What types of plasma processes are triggered by magnetosheath jets? How often are these processes triggered? What is the link between jets and reconnection, surface and body waves, field-aligned currents, ionospheric vortices, and ground magnetic field perturbations? Can different properties of jets trigger different processes? How are these triggered? For example, what triggers particle acceleration that causes auroral brightening? Bursty reconnection or ULF waves or another process? How is momentum and energy transferred through these processes? Where is the energy deposited? What physical processes are most important for energy and momentum transfer into the magnetosphere?

Establishing a connection between an impacting jet and the triggered physical process is challenging due to limited points of observations. Conjugations between different missions may be a useful tool to track the effects of jets. The use of different ground instrumentation, such as radar systems, all-sky cameras, and magnetometers is needed.

**What jet properties influence the geoeffectiveness of jets?**

How do jet properties, such as dynamic pressure and magnetic field, affect their geoeffectiveness? What properties of jets are significant? Are single impacting jets or trains of jets more important? What are the spatial and temporal scales involved and how do they affect the significance?

The above questions require statistical studies for processes that have already been identified. In recent years, jets have been classified in different groups, depending on the occurrence in the quasi-parallel or quasi-perpendicular magnetosheath, or their association with a rotation in the IMF (Raptis et al. 2020b). These jets are likely to have different properties due to different formation mechanisms. How do these different properties drive their geoeffectiveness?

### Future Missions

We will briefly highlight how future plasma missions at Earth can be used for research concerning magnetosheath jets. Particularly, the missions HelioSwarm, SMILE, and Plasma Observatory are of interest for jet research.

HelioSwarm is a NASA medium sized explorer (MidEx) designed to study plasma turbulence and three dimensional processes in the solar wind (Klein et al. 2019, 2023). The mission consists of nine spacecraft; one hub spacecraft, and eight daughter spacecraft. The hub spacecraft measures the magnetic field with fluxgate and search coil magnetometers, and the plasma properties with Faraday cups and an ion electrostatic analyser. The daughter spacecraft only carries magnetometers and Faraday cups. Out of the total projected measurements taken by HelioSwarm, 35% are expected to be in the magnetosheath or magnetosphere. While the plasma instruments are optimised to measure the cold solar wind, rather than the hotter magnetosheath plasma, the multi-point magnetometer data may give valuable insight into magnetosheath jets.

SMILE (estimated to be launched June 2025) is an upcoming mission from the European Space Agency (ESA) and the Chinese Academy of Sciences (CAS) (Branduardi-Raymont and Wang 2024). One of the main objectives of the SMILE mission is to understand the dayside solar wind-magnetosphere interaction. The Soft X-ray Imager (SXI) on-board will harness the process of solar wind charge exchange to image the magnetosphere in soft X-rays. This process will hopefully provide remote-sensing observations on boundary locations of both the bow shock and the magnetopause (Sibeck et al. 2018, June). Interpreting SMILE data will require dedicated modelling efforts. Already, several articles using hybrid simulations predict that magnetosheath jets and their interaction with the Earth’s magnetopause should modulate X-ray intensities which might be observable by SMILE (see, e.g., Guo et al. 2023; Yang et al. 2024). However, expected numbers of counts over the spatial and temporal scales of jets’ magnetospheric impacts are likely low, and hence it is still unclear if the SXI instrument will be able to resolve these physical signals (Samsonov et al. 2022; Ng et al. 2023).

Plasma Observatory (Retinò et al. 2023) is at an earlier mission phase than HelioSwarm and SMILE. At this point, it is still a candidate M-class mission of ESA, currently competing in phase A against Theseus (Amati et al. 2018) and M-MATISSE (see below) (Sanchez-Cano et al. 2022). The main goals of the mission are to unravel how particles are energised in space plasmas and to determine which processes contribute most to energy transport across scales and magnetospheric regions. The mission shall consist of a fleet of seven spacecraft in total: one mothercraft (MSC) and up to 6 additional and identical small satellites, called daughtercraft (DSC). The spacecraft shall fly through all main magnetospheric regions around Earth, ideally in a configuration resembling two nested tetrahedra. Therefore, it would be possible to provide valuable observations on cross-scale energy coupling. Plasma jets as energy transport processes are explicit targets of observation of the proposed mission.

In addition, several missions to other planetary bodies are planned which are potentially useful to study magnetosheath jets. This would allow for the demonstration of the universality of shock processes.

BepiColombo is a two-spacecraft mission to Mercury which will arrive in December 2025 (Benkhoff et al. 2010). The magnetospheric orbiter, Mio, has a full plasma instrument payload, and a suitable orbit to study magnetosheath jets in the Mercury environment. Ion density and velocity moments will be available with spin resolution (4 s). For higher time resolution, the $\mathbf{E}\times \mathbf{B}$ drift velocity can be determined from the electric and magnetic field measurements, as well as density determined by the spacecraft potential. This should enable measurements of magnetosheath jets if they exist at Mercury.

Similar two-spacecraft measurements may be available from the Mars environment in the future, should the Mars Magnetosphere ATmosphere Ionosphere and Surface SciencE (M-MATISSE) M-class mission candidate be selected by ESA (Sanchez-Cano et al. 2022). If so, the mission would be launched in 2037 (Sánchez-Cano 2023). The main goal of the mission will be to investigate the dynamic response of the coupled Martian magnetosphere-atmosphere-surface environment to inputs from the solar wind and solar radiation. While a primary interest of the mission is to understand space weather at Mars in preparation for future human exploration, the spacecraft should be fully equipped to measure plasma particle and field quantities, allowing the study of phenomena like magnetosheath jets with multi-point observations.

## Concluding Words

Magnetosheath jets have gained much attention over the last 25 years. We have reviewed findings on magnetosheath jets from the last five years, during which significant progress has been made. Additional jet generation mechanisms have been identified and solar wind parameters that enhance or suppress jet formation have been found. In addition, jets have now been shown to exist in other planetary and shock environments making them a phenomenon present across the solar system. The evolution of magnetosheath jets and how they interact with the ambient plasma has also been studied to a significant degree. In particular, the evolution of jets’ properties and the disruption they cause to magnetosheath and magnetopause is now apparent. Global simulations have been found to be a useful tool to investigate jets as they propagate through the magnetosheath. This has allowed us to evaluate the evolution of their properties across multiple scales, while providing a global picture of how their formation is connected to bow shock dynamics.

Though several of the open questions raised by Plaschke et al. (2018) have been addressed, many new points of intrigue have emerged. The need to investigate magnetosheath jets continues as their place as a fundamental process of collisionless shocks becomes more apparent. In the future, upcoming missions will provide exciting opportunities to study jets at other planets and shock environments as well as on different scales in the terrestrial magnetosheath. We conclude this review by inviting the reader to join the efforts to understand the causes and effects of magnetosheath jets.
